# Synthesis Candidates Herbicide Through Optimization Quinclorac Containing 3-Methyl-1H-pyrazol-5-yl

**DOI:** 10.3389/fchem.2021.647472

**Published:** 2021-04-14

**Authors:** Dingfeng Luo, Haodong Bai, Xiaomao Zhou, Lamei Wu, Chengjia Zhang, Zhongchi Wu, Zuren Li, Lianyang Bai

**Affiliations:** ^1^ Long Ping Branch, Graduate School of Hunan University, Changsha, China; ^2^ Hunan Provincial Key Laboratory for Biology and Control of Weeds, Hunan Academy of Agricultural Sciences, Changsha, China

**Keywords:** quinclorac, intermediate derivatization methods (IDMs), barnyard grass, herbicidal activity, field assay

## Abstract

To enhance quinclorac potency, twenty-five derivatives were synthesized containing 3-methyl-1H-pyrazol-5-yl by intermediate derivatization methods (IDMs). These compounds were confirmed by melting point (mp), ^1^HNMR, ^13^CNMR, and HRMS. The compound 1,3-dimethyl-1H-pyrazol-5-yl 3,7-dichloroquinoline-8-carboxylate (10a) was determined by X-ray diffraction. The activity of these compounds substituent on the phenyl was: electron-drawing group > neutral group > donor-drawing group, the results was like that of substituted benzyl group on pyrazole. The herbicidal activity assays showed that compounds 1-(2-fluorophenyl)-3-methyl-1H-pyrazol-5-yl 3,7-dichloroquinoline-8-carboxylate (8l, EC_50_ = 10.53 g/ha) and 10a (EC_50_ = 10.37 g/ha) had an excellent inhibition effect on barnyard grass in greenhouse experiment. Greenhouse safety experiment of rice exhibited almost no difference in plant height and fresh weight treated 10a at stage 1∼2-leaf of rice after 14 days but 8l had a detrimental effect. Two season field assays showed 10a herbicidal activity on barnyard grass at 150 g/ha as equal as 300 g/ha quinclorac in fields in 2019 and 2020. The study demonstrated that 10a could be further researched as a potential herbicide to control barnyard grass in fields.

## Introduction

Pyrazole-containing compounds have played an important role in the development of heterocyclic agrochemicals because the pyrazole structure enables multidirectional transformations, and the introduction of different substituents on pyrazole provides a diversity of structures (Fu et al., 2017; Jiao et al., 2019; Fu et al., 2020). Furthermore, as pyrazole-containing compounds exhibit various biological activities, high selectivities, and low toxicities, they have received attention for the development of new pesticides in recent years (Du et al., 2015; Lv et al., 2015; Yao et al., 2017; Zhao et al., 2019), for example, pyrazosulfuron, azimsulfuron, benzofenap, and pyrazolate. Several pyrazole-containing agriculture chemicals have been commercialized. Fipronil, as an inhibitor of GABA–chloride ion channel, has a good control effect on pests resistant to organophosphorus, organochlorine, and pyrethroid and other insecticides, and it can be used to control Lepidoptera and Coleoptera pests of vegetables, rice, and cotton (Burris et al., 1994; Gaulliard, 1996; Shaw et al., 1997). Several pyrazole-containing derivatives with high insecticidal activity are developed and successfully marketed by using fipronil as a lead compound such as acetonitrile and butenofluronitrile. Pyraclostrobin exhibits excellent bactericidal activity and has a remarkable control effect on cucumber downy mildew, early blight of tomato, cucumber anthracnose, and so on (Hou et al., 2002; Karadimos et al., 2005; Qiao et al., 2009). Pyrazolynate was the first significant 4-hydroxyphenyl-pyruvate dioxygenase (HPPD) herbicide containing a pyrazole ring to enter the herbicide market, although the action target site was then unknown (Konotsune et al., 1975; Nakagawa et al., 1988; Cole et al., 2000).

The intermediate derivatization methods (IDMs), which consist of three approaches, namely, the common intermediate method (CIM), the terminal group replacement method (TRM), and the active compound derivatization method (ADM), have been widely used to innovate new agricultural compounds (Guan et al., 2014; Guan et al., 2017). For example, [2-(9H-carbazol-9-yl) ethanol-11-oxoundecyl] pyridinium bromide was synthesized and exhibited approximately increased 19-fold enhancement in the anti-*Ralstonia solanacearum* efficacy (Wang et al., 2017). Compound SYP-9121 was synthesized using IDMs, which showed a broad-spectrum herbicidal activity in greenhouse and herbicidal activity being comparable mesotrione, good selectivity, and safety in maize field trials (Li et al., 2017). IDMs were an effective way to modify agricultural compounds by enhancing their bioactivity.

**FIGURE 1:**
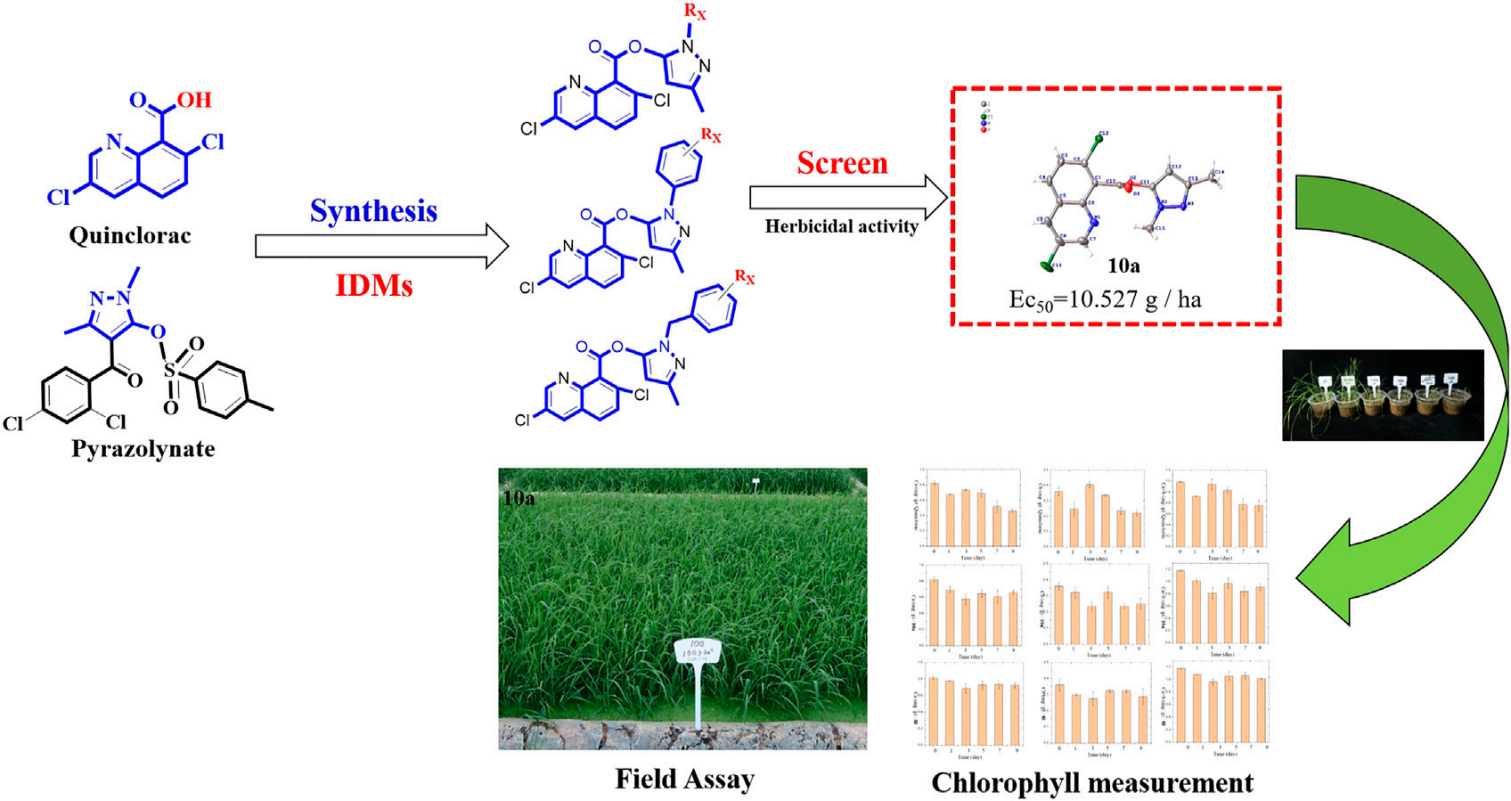
Design of the target compounds.

Quinclorac was a special selective herbicide for controlling barnyard grass in paddy fields (Kießling and Pfenning, 1990; Grossmann and Kwiatkowski, 1993; Grossmann and Kwiatkowski, 2000). Quinclorac was chosen as a vital intermediate to be modified using IDMs in order to improve the herbicidal activity or innovate new herbicide (Figure 1). A series of novel quinclorac–pyrazole derivatives were designed, synthesized, and characterized, and the herbicidal activity against gramineous weeds, barnyard grass, tall oatgrass (*Arrhenatherum elatius* (L.) Presl.), dicotyledon weeds*,* romaine lettuce (*Lactuca sativa* var. longifolia. Lam.), and lettuce (*Lactuca sativa L.* var. *ramosa* Hort*.*) was greenhouse-tested.

## Materials and Methods

### Instruments and Materials

All reagents and chemicals were purchased from commercial sources, including 2,5-dimethyl-2,4-dihydro-3H-pyrazol-3-one (**2o**). ^1^H and ^13^C nuclear magnetic resonance (NMR) spectra were recorded using a Bruker Avance-400 spectrometer (*Bruker* BioSpin AG, Fällanden, Switzerland). Deuterated chloroform (CDCl_3_) and deuterated dimethyl sulfoxide (DMSO-*d*_6_) were used as NMR solvents and tetramethylsilane (TMS) was used as an internal standard. Melting points (mp) were recorded on the Hanon MP100 melting point apparatus (Hanon Instruments, Jinan, China). High-resolution mass spectral analysis was carried out using a Varian 7.0 T FTICR-MS instrument (Varian IonSpec, Lake Forest, CA, United States). Single-crystal X-ray data were obtained using a Bruker SMART APEX II X-ray single-crystal diffractometer (Bruker AXS, Karlsruhe, Germany), graphite monochromated Mo Kα radiation (λ = 0.71073 Å), a 3WP-2000-Spray tower (Nanjing Institute of Agricultural Mechanization, Ministry of Agriculture and Rural Affairs, Nanjing, China), and a A560-UV-VIS spectrophotometer (Aoyi Instruments Co., Ltd., Shanghai, China).

### General Procedure for Preparing the Intermediates of 2a-n

Methyl acetoacetate (25 mmol) was added to 20 ml of acetic acid containing different substituted phenylhydrazine hydrochloride (25 mmol) and sodium acetate (26 mmol) in nitrogen atmosphere. The reaction mixture could reflux with stirring overnight. The reaction solution was poured into 50 ml ice-cold water, and the mixture was adjusted to pH 7.0–8.0 using saturated NaHCO_3_ solution. Subsequently, the mixture was extracted with ethyl acetate (3 × 50 ml). The combined ethyl acetate was dried over anhydrous MgSO_4_ and then concentrated to obtain the crude product. Purification by column chromatography (silica gel, 6:1 hexane/ethyl acetate) gave product 2a-n. The characterization data for the intermediates of 2a-n are shown in Supplementary Material.

### 5‐Methyl‐2‐phenyl-2,4-dihydro-3H-pyrazol-3-one (2a)

Yellow solid; yield: 73%, mp: 130.7–131°C; ^1^H NMR (400 MHz, CDCl_3_, δ ppm): δ 7.85 (d, *J* = 7.6 Hz, 2H, Ar-H), 7.38 (*t*, *J* = 7.6 Hz, 2H, Ar-H), 7.17 (*t*, *J* = 6.8 Hz, 1H, Ar-H), 3.43 (d, *J* = 3.6 Hz, 2H, CH_2_), 2.18 (d, *J* = 4 Hz, CH_3_); ^13^C NMR (100 MHz, CDCl_3_, δ ppm): δ 170.57, 156.33, 138.02, 128.83, 125.04, 118.87, 43.12, and 17.05. HRMS (ESI) C_10_H_10_N_2_O [M + H]^+^: calcd.175.0866, found 175.0862.

### General Procedure for Preparing the Intermediates of 5a-j

5-Methyl-methyl-2,4-dihydro-3*H*-pyrazol-3-one 3 (10 mmol) and different substituted benzyl bromide (4a-i) or ethyl bromide (12 mmol) were added to 1,4-dioxane (120 ml) and refluxed. After 48 h, the reaction mixture was filtered, the solvent was evaporated, and 10% NaHCO_3_ solution was added. Subsequently, the mixture was extracted with ethyl acetate (3 × 100 ml). The combined ethyl acetate was washed with salt water and dried over anhydrous MgSO_4_, and then rotary evaporation under reduced press obtained the raw product. Recrystallization from ethanol/*n*-hexane (1 ː 3) gave product 5a-j. The characterization data for the intermediates of 5a-j are shown in Supplementary Material.

### 2-Benzyl-3-methyl-1H-pyrazol-5-ol (5a)

White solid; yields: 71%, mp: 160.3–160.8°C; ^1^H NMR (400 MHz, CDCl_3_, δ ppm): δ 7.30 (m, 2H, Ar-H), 7.23–7.27 (m, 1H, Ar-H), 7.13 (d, *J* = 8.4 Hz, 2H, Ar-H), 5.43 (s, 1H, CH), 5.06 (s, 2H, Ar-CH_2_), and 2.13 (s, 3H, N-C-CH_3_); ^13^C NMR (100 MHz, CDCl_3_, δ ppm): δ 161.55, 140.47, 136.87, 128.75, 127.58, 126.73, 91.47, 51.93, and 11.38; HRMS (ESI) C_11_H_12_N_2_O [M-H]^-^: calcd.187.0877, found 187.0874.

### General Procedure for Preparing the Target Compounds 8a–n, 9a–i, and 10a–b.

Quinclorac 6 (3.31 mmol) dissolved in 50 ml SOCl_2_, refluxed for 10 h. Then, the solution was distilled under reduced pressure to obtain crude 3,7-dichloroquinoline-8-carbonyl chloride 7, which was used in the next step without further purification.

Different intermediates of 2a-o and 5a-j (10 mmol), calcium oxide (6 mmol), calcium hydroxide (14 mmol), and 1,4-dioxane (100 ml) were added to a two-neck flask and refluxed for 10–20 min. Then, 3,7-dichloroquinoline-8-carbonyl chloride 7 (12 mmol) in 1,4-dioxane (20 ml) was added, and the obtained mixture was refluxed for a further 4–5 h. Then, the reaction solution was poured into ice-cold water, and 20 ml of 4 M HCl solution was added to the mixture. Subsequently, the solution was extracted with ethyl acetate (3 × 60 ml). The mixture was washed three times with 100 ml brine water, dried over anhydrous MgSO_4_, and then concentrated to obtain the product. Purification using column chromatography (silica gel, hexane/ethyl acetate = 3:1) gave the product. The characterization data for the compounds 8a–n, 9a–i, and 10a–b are shown in Supplementary Material.

### 3-Methyl-1-phenyl-1H-pyrazol-5-yl 3,7-dichloroquinoline-8-carboxylate (8a)

Light yellow solid; yield: 62%; mp: 135.7–135.9°C; ^1^H NMR (400 MHz, CDCl_3_, δ ppm): δ 8.80 (d, *J* = 1.6 Hz, 1H, Quinoline-H), 8.16 (d, *J* = 2.0 Hz,1H, Quinoline-H), 7.80 (d, *J* = 8.8 Hz, 1H, Quinoline-H), 7.63–7.65 (m, 2H, Ar-H), 7.59 (d, *J* = 8.8 Hz, 1H, Quinoline-H), 7.32–7.36 (m, 2H, Ar-H), 7.26–7.28 (m, 1H, Ar-H), 6.47 (s, 1H, CH), and 2.40 (s, 3H, N-C-CH_3_): ^13^C NMR (100 MHz, CDCl_3_, δ ppm): δ161.28, 151.24, 149.04, 144.08, 143.60, 137.91, 133.84, 132.48, 131.09, 129.83, 128.82, 127.06, 126.57, 123.57, 96.11, and 14.58; HRMS (ESI) C_20_H_13_N_3_O_2_Cl_2_ [M + H]^+^: calcd. 398.0458, found 398.0459.

### X-Ray Diffraction of Compound 10a

A suitable single crystal (0.13 × 0.12 × 0.1 mm) of 10a was obtained by recrystallization from ethanol. The cell dimensions and intensities were measured at 100 K on Bruker SMART APEX II X-ray single-crystal diffractometer using graphite monochromator *Mo-K*α radiation (λ = 0.71073 Å); θmax = 49.99, 4,865 measured reflections, and 2,624 independent reflections (R_int_ = 0.0467). The structure was solved by the direct method and refined using *SHELXS-97* by full-matrix least squares on F^2^ using the weight of *ω* = 1/[σ^2^ $\left(F_{o}^{2}\right)$ + (0.0702 P)^2^ + 0.0736 P] and gave final values of *R* = 0.0498, *ωR*_2_ = 0.1213, max/min residual electron density = 0.29, and−0.32 e. Å^−3^. (Sheldrick, 2008; Yüksektepe et al., 2010; Fu et al., 2017). The crystallographic data of compound 10a were deposited with the Cambridge Crystallographic Data Center (CCDC) under deposition no. 2022300. The detailed information on the structure of 10a compound could be downloaded free via http://www.ccdc.cam.ac.uk/.

**FIGURE 2:**
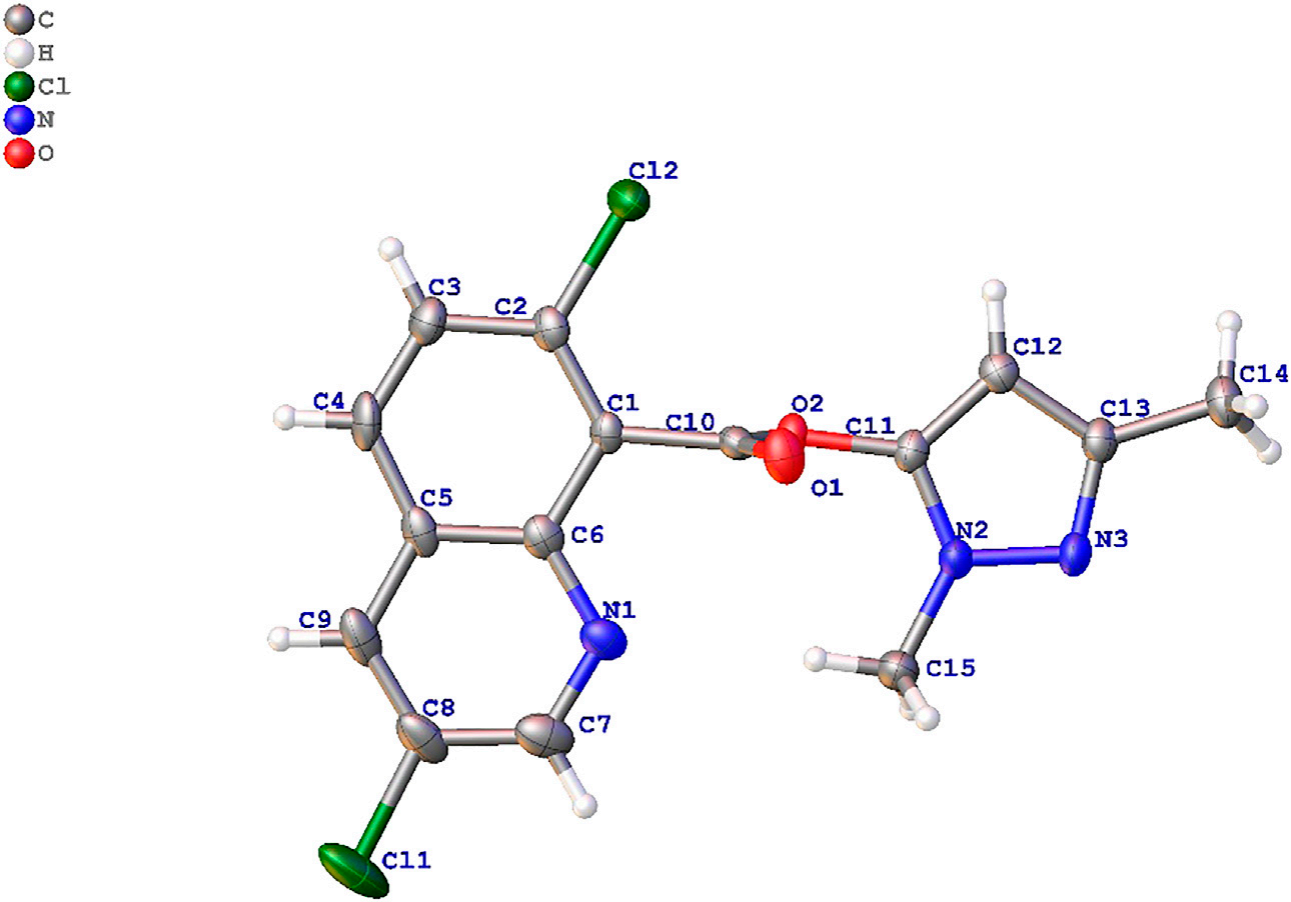
Crystal structure of compound 10a.

Compound 10a is composed of three rings, and the bond lengths and bond angles are in the usual ranges in crystal structure of 10a in Figure 2.

### Biological Assays

Herbicidal activities of the compounds on monocotyledon weeds (barnyard grass and tall oatgrass) and dicotyledon weeds (romaine lettuce and lettuce) were determined in greenhouse. Compounds were formulated as 100 g/L emulsified concentrates by using dimethylformamide as the solvent and Tween-80 as the emulsification reagent. The concentrates were diluted with water to obtain the required concentration, and the concentration of Tween-80 was kept at 0.1%. Plastic pots with a diameter of 9.5 cm were filled with soil to a depth of 4.5 cm containing 33.3% vermiculite and 66.7% nursery substrates. The seeds of the test plants were sown separately according to the species. 12–15 seeds of the tested weeds were sown in the soil at a depth of 0.2 cm and grown at 18–35°C in a glasshouse. The monocotyledon weeds were treated at the 1∼3-leaf stage, and dicotyledon weeds were treated at the 2∼4-leaf stage. Untreated weeds with the mixture of the same amount of water, dimethylformamide, and Tween 80 were sprayed as blank control. All tests were repeated three times. Visual injury herbicidal activity was investigated at 14 days after treatment (DAT). Evaluation was on a scale from 0 to 100, where 0 meant no damage or normal growth and 100 meant no emergence of the plants or complete destruction of at least aerial parts (Li et al., 2006; Chen et al., 2009). Quinclorac was selected as treated control. The term half maximal effective concentration (Ec_50_) was calculated by the inhiation rate of barnyard grass under the application rate of 3.75, 7.5, 15, 30, 60, and 120 g a.i./ha were sprayed on barnyard grass. All tests were repeated in three times.

Safety experiment of rice was carried out at a dosage of 375 g a.i./ha of compounds 8l and 10a at a 1∼2-leaf stage, which was investigated at 14 DAT through measuring the fresh weight and plant height of rice aerial parts.

The chlorophyll (chlorophyll a (C_a_), chlorophyll b (C_b_), and total chlorophyll (C_a+b_)) levels were surveyed after drug application 0–9 days and evaluated with three duplicates per experiment, and the procedure was repeated three times (Wellburn, 1994). A bulk extract of chlorophyll from the grass leaves was made in semi-darkness using the following formulation: 95 : 5, v/v/, acetone/ethanol. The contents of chlorophyll a (C_a_) and chlorophyll b (C_b_) were determined by Eqs 1 and 2, respectively:

$${\text{C}}_{\text{a}}=\left({\text{A}}_{663}\times 12.72-{\text{A}}_{645}\times 2.59\right)\times \text{V}/\left(\text{W}\times 1000\right),$$ (1)

$${\text{C}}_{\text{b}}=\left({\text{A}}_{663}\times 22.88-{\text{A}}_{645}\times 4.68\right)\times \text{V}/\left(\text{W}\times 1000\right),$$ (2)

where Ca and C_b_ are the contents of chlorophyll a and chlorophyll b (mg/g), respectively’ A_645_ and A_663_ are optical densities at 645 and 663 nm, respectively; V is the constant volume (ml); and W is the weight of the sample (g).

Field assays were conducted according to the field efficacy trials of Chinese pesticide standards (GB/T17980.40–2000) in Changsha, Hunan Province, from June to August 2019 and 2020. All concentrations set three experimental plot and each plot twenty square meters in field assays. The test weeds contained barnyard grass as a typical monocotyledon weed. The concentrations of the target compounds were 45, 75, 120, and 150 g a.i./ha (5% emulsifiable concentrate), untreated plot with the mixture of the same amount of water, dimethylformamide, and Tween 80 were sprayed as blank control and quinclorac was used as treated control at a concentration of 300 g a.i./ha. The test weeds in the rice fields were at the 1∼2-leaf stage and the rice was at the 1∼2-leaf stage.

Control effect (%) = (CK-PT)/CK × 100, CK means blank control plots weeds number, PT means compound 10a and quinclorac treatment plots weeds plants number.

Data was analyzed using DPS and variance (ANOVA) (Tang and Zhang, 2013).

## Results and Discussion

### Synthetic Chemistry

**FIGURE 3:**
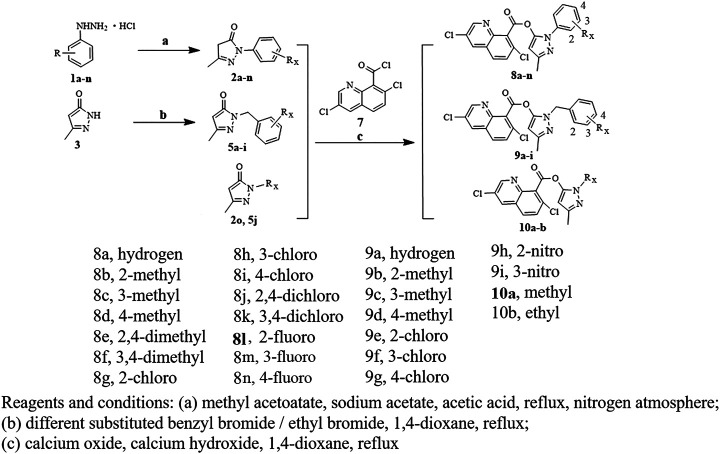
Synthesis route to target compounds 8a-n, 9a-i, and 10a-b.

The synthetic scheme was designed to synthesize target compounds 8a-n, 9a-i, and 10a-b, which were dependent on substituted R_x_ ( Figure 3). It was found that target compounds 8a-n, 9a-i, and 10a-b were created by three-step reactions containing the precursor synthesis. The precursors 2a-m and 5a-k were synthesized according to the protocol reported previously (Belmar et al., 2001; Sheng et al., 2015; Han et al., 2016). Methyl acetoacetate, different substituted phenylhydrochloride, and sodium acetate in acetic acid solution heated to reflux synthesized 2a-m in an inert gas atmosphere, which was achieved in 41–80% yields. 5-Methyl-2,4-dihydro-3H-pyrazol-3-one and ethyl bromide or different substituted benzylbromide were heated to reflux in 1,4-dioxane solution, which synthesized precursor 5a-k with 38–71% yields. There are 5-methyl-2-phenyl-2,4-dihydro-3H-pyrazol-3-one derivatives of enol type and ketone type in the molecule (Nakagawa et al., 2006). This is shown in precursors 2b, 5a, 5b, 5f, 5h, and 5j. Precursor 3,7-dichloroquinoline-8-carbonyl chloride was heated with quinclorac in thionyl chloride solution without purification, which is used in the next step.

Target compounds 8a-n, 9a-I, and 10a-b were synthesized *via* the precursor 2a-n and 5a-j and 3,7-dichloroquinoline-8-carbonyl chloride in the presence of calcium hydroxide and calcium oxide in the 1,4-dioxane solution in 51–76% yields.

### Herbicidal Activity in Greenhouse Experiments

The postemergence herbicidal activities of the synthesized compounds for four kinds of weeds were evaluated using greenhouse experiments with quinclorac as a positive control (Table 1). At a concentration of 375 g a.i./ha, four of the target compounds (8l, 9h, 9i, and 10a) had the same effect as quinclorac on controlling barnyard grass. In addition, compounds 8l could cause local green loss of barnyard grass leaves at low concentration. The target compounds and quinclorac had little effect on tall oatgrass. The inhibitory effects of five target compounds (8f, 8k, 8l, 9g, and 9i) on romaine lettuce were like those of quinclorac at a concentration of 375 g a.i./ha. All the synthesized compounds influenced lettuce, with half showing the same effect as quinclorac and the others displaying > 40% inhibition. When pyrazole was substituted the phenyl group, compounds with neutral chlorine atom had better weeding activity on barnyard grass than the electron-donating methyl with the same position except meta-position, but all had poor weeding activity. Electron-drawing group fluorine atom substituted on benzene was more effective than the neutral atom; ortho-position compound 8l had an equal effect to quinclorac. The herbicidal activity of these compounds substituent group on the phenyl was: electron-drawing group > neutral group > donor-drawing group. When pyrazole has substituted benzyl group has similar changes with herbicidal activity on barnyard grass (electron-drawing group > neutral group > donor-drawing group).

**TABLE 1:** Postemergence herbicidal inhibition activity of compounds 8a-n, 9a-I, and 10a-b at a dosage of 375 g a.i./ha.

Com	R_x_	Inhibition (%)^a^			
		E.c.	A.e.	V.r.	L.h.
8a	H	**+++**	−	++	+++
8b	2-CH_3_	**+**	+	+	+++
8c	3-CH_3_	**+++**	−	+++	++++
8d	4-CH_3_	**+**	+	+	+++
8e	2,4-CH_3_	**-**	+	++++	+++++
8f	3,4-CH_3_	**++**	−	+++++	+++++
8g	2-Cl	**++**	+	+	+++
8h	3-Cl	**+**	−	+	+++
8i	4-Cl	**+++**	++	++++	+++++
8j	2,4-Cl	**+++**	−	+++	++++
8k	3,4-Cl	**+++**	−	+++++	+++++
8l	2-F	**+++++**	+	+++++	+++++
8m	3-F	**+++**	+	++++	+++++
8n	4-F	**+++**	+	+++	+++++
9a	H	**+**	−	+	++++
9b	2-CH_3_	**++**	−	+	++++
9c	3-CH_3_	**++**	−	+	++++
9d	4-CH_3_	**+**	−	+	+++
9e	2-Cl	**++**	−	+	+++++
9f	3-Cl	**+**	+	++	++++
9g	4-Cl	**+**	−	+++++	+++++
9h	2-NO_2_	**+++++**	+	+++	++++
9i	3-NO_2_	**+++++**	+	+++++	++++
10a	CH_3_	**+++++**	+	++	+++++
10b	CH_2_CH_3_	**+++**	+	+++	+++++
Quinclorac	−	**+++++**	−	+++++	+++++

^a^ Rating scale of herbicidal activity (percentage of inhibition): +++++, ≥ 95%; ++++, > 70%; +++, 40–70%; ++, 20–40%; +, 10–20%; −, < 10%. E.c.: barnyard grass, A.e.: tall oatgrass, V.r.: romaine lettuce, and L.h., lettuce.

Compounds 8l and 10a, which showed favorable herbicidal activities against barnyard grass, were selected for further evaluation and the EC_50_ values were determined (Table 2). The CLogP value of compounds 8l and 10a calculated by ChemDraw (CambridgeSoft) was 5.42 and 3.27, respectively, which meant that 10a had better solubility in water than 8l. The solubility of compound 10a in water was better than that of compound 8l in the experiment of concentration gradient of barnyard grass. They were selected for testing the efficacy of resistance barnyard grass because the resistance of quinclorac became higher in the Hunan area (Ma et al., 2012). However, they caused some damage to the resistant barnyard grass but could not kill them at the concentration of 375 g a.i./ha. Therefore, they might have a similar pathway of action on barnyard grass with quinclorac (Yasuor et al., 2012).

**TABLE 2:** EC_50_ values of compounds 8l and 10a against barnyard grass.

Com	EC_50_ (g·ha^−1^)	95% fiducial limits	Slope ± SE	Chi-square
8l	10.53	6.31–15.70	3.00 ± 0.25	12.76
10a	10.37	6.50–14.78	2.26 ± 0.21	6.79

### Chlorophyll Content Measurement

**FIGURE 4:**
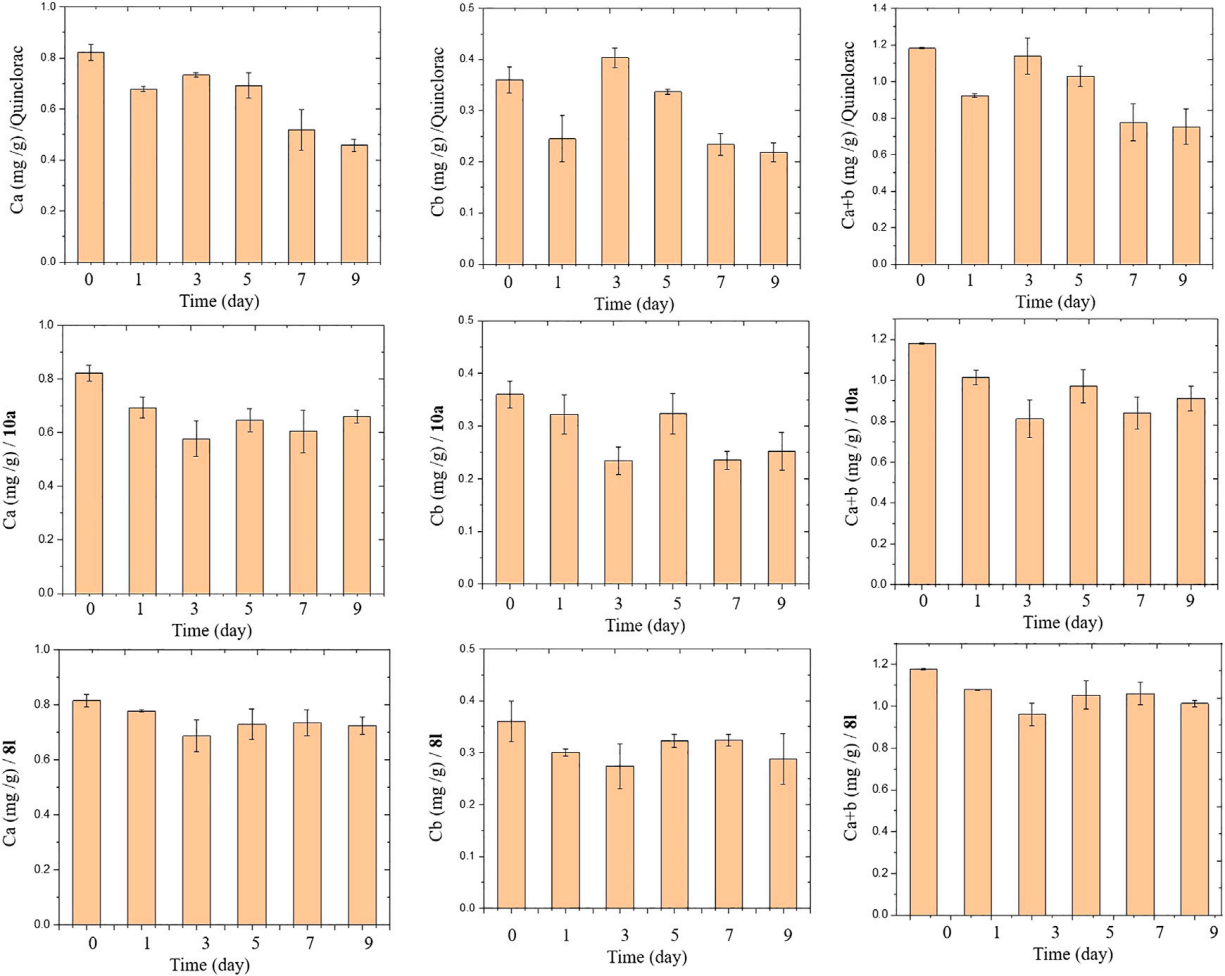
The leaf chlorophyll a, b, and a + b contents at 2∼3 stages in barnyard grass seedling treated with quinclorac and compounds 8l and 10a during 0–9 days. Data are the mean + standard errors (SE).

Leaf chlorophyll allows plants to absorb energy from light to support photosynthetic production. However, compounds 8l and 10a had favorable herbicidal activities against barnyard grass and 8l caused a local loss of green at low concentrations. The change in leaf chlorophyll content of barnyard grass in response to treatment with compounds 8l, 10a, and quinclorac is shown in Figure 4. The general trend of chlorophyll a, b, and a + b contents decreased sprayed with 8l, 10a and quinclorac 375 g a.i./ha contrast to untreated after nine days. The general trend of chlorophyll a, b, and a + b contents treated 10a and quinclorac are consistent, their content down after treated then up and down again but quinclorac go up on the third day and 10a on the fifth day. The chlorophyll a, b, and a + b contents of compound 8l are similar with 10a but contents variation is not as great as it.

### Greenhouse Safety Experiment of Rice

**FIGURE 5:**
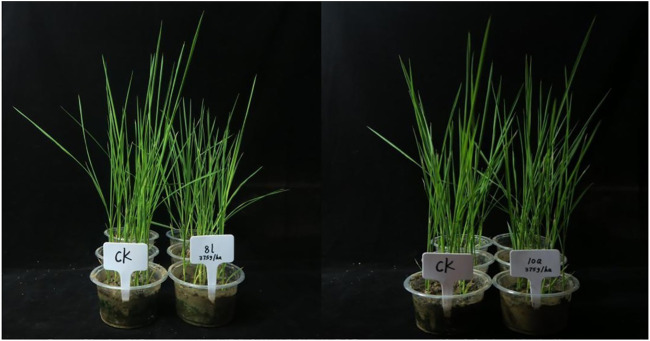
Rice safety experiment in greenhouse-treated 375 g a.i./ha 8l and 10a after 14 days.

As compounds 8l and 10a displayed strong inhibitory effects on barnyard grass at a dosage of 150 g a.i./ha, they were selected for further rice safety experiments in the greenhouse. For the safety studies, rice at the 1∼2-leaf stage was treated at a dosage of 375 g a.i./ha. After 14 days, the average plant height of rice treated with 8l was 86.01% of that of the untreated control and the average fresh weight was 81.60% of that of the untreated control, which indicated that 8l had some phytotoxic effect on rice. When treated with 10a, after 14 days, the average plant height was 98.7% of that of the untreated control and the average fresh weight was 96.1% of that of the untreated control. Thus, 10a had almost no phytotoxic effect on rice (Figure 5), which indicated the potential of 10a for further field trials as a herbicide candidate to control weeds in rice paddy.

### Herbicidal Activities in Field Assay

The weeding effect of the field assay was investigated at different concentrations of 10a on 14, 21, and 52 days, respectively, after application in 2019 and 2020. The concentration of compound 10a was 45, 75, 120, and 150 g a.i./ha (5% EC (emulsifiable concentrate)), and quinclorac was used as the positive control at a concentration of 300 g a.i./ha. The control effect of compound 10a increased with the increase in its concentration. The weeding effect was better on 21 days than 14 days with the same concentration of compound 10a, and it was worse on day 52 than on day 14. The control effect of barnyard grass was 40.7–89.2% and 52.4–94.5% after 14 and 21 days treated compound 10a at a concentration from 45 to 150 g a.i./ha at 2019. The highest concentration of 10a herbicidal effect was 94.5%, equal to 300 g a.i./ha of quinclorac against barnyard grass. The similar result was present in 2020. The field test results were shown in Table 3 and in Supplementary Figure S1. But compound 10a was not very effective in the control of broad-leaved grass such as *Monochoria vaginalis* and *Ludwigia prostrata* Roxb at a dosage of 150 g a.i./ha in rice field. All these suggested that the 10a compound was a potential herbicide for further development as controlling barnyard grass in rice fields.

**TABLE 3:** Weed control effects after 14 and 21 days of treatment at different dosages of 10a and quinclorac.

Treatment g/ha	Barnyard grass (control effect %)				Total weeds (control effect %)			
	2019		2020		2019		2020	
	14 days	21 days	14 days	21 days	14 days	21 days	14 days	21 days
45 (10a)	40.7 ± 1.67d	52.4 ± 3.36d	43.7 ± 2.07d	50.6 ± 1.36d	40.1 ± 2.81d	45.6 ± 0.51d	38.8 ± 0.70d	44.9 ± 2.10d
75 (10a)	65.1 ± 0.68c	76.1 ± 0.95c	68.9 ± 1.53c	74.0 ± 1.27c	65.2 ± 0.37c	70.4 ± 1.67c	62.3 ± 1.91c	68.1 ± 0.51c
120 (10a)	78.6 ± 2.93b	84.0 ± 1.73b	79.8 ± 0.83b	83.4 ± 1.10b	77.8 ± 0.57b	81.9 ± 0.44b	78.9 ± 0.21b	83.2 ± 0.45b
150 (10a)	89.2 ± 0.56a	94.5 ± 0.71a	89.6 ± 1.09a	93.5 ± 0.20a	89.7 ± 0.92a	91.6 ± 1.00a	92.7 ± 0.27a	95.2 ± 0.85a
300 (Q)	91.8 ± 1.30a	94.1 ± 0.73a	90.2 ± 0.93a	93.8 ± 1.70a	90.0 ± 1.25a	92.3 ± 0.50a	91.8 ± 0.61a	94.0 ± 0.50a

Data analysis is based on the average of three repetitions. Q: quinclorac. The different letters indicate the significant difference according to the SNK test (α = 0.05).

## Conclusion

A series of novel quinclorac–pyrazole derivatives were designed and synthesized containing 3-methyl-1H-pyrazol-5-yl using quinclorac as the starting material through IDMs and were characterized by mp, ^1^HNMR, ^13^CNMR, and HRMS. The herbicidal activity of these compounds substituent on the phenyl having electron-drawing group was greater than the neutral group, and the neutral group was greater than the donor-drawing group; the results were similar to those of the substituted benzyl group on pyrazole. Compounds 8l (EC_50_ = 10.53 g/ha) and 10a (EC_50_ = 10.37 g/ha) had excellent inhibitory effects on barnyard grass in a greenhouse and compound 10a exhibited almost no phytotoxic effect in rice safety experiment. Field assays showed that 10a has a good herbicidal effect on barnyard grass but a little effect on broad-leaf grass. Research work for further optimization of the potential herbicide 10a is in progress.

## Data Availability

The datasets presented in this study can be found in online repositories. The names of the repository/repositories and accession number(s) can be found in the article/Supplementary Material.
