# Supplementary material for: Synthesis Candidates Herbicide Through Optimization Quinclorac Containing 3-Methyl-1H-pyrazol-5-yl
Source: Front Chem. 2021 Apr 14;9:647472. doi: 10.3389/fchem.2021.647472 (PMC8080966; doi:10.3389/fchem.2021.647472)
Supplement: Supplementary file 1 [file datasheet1.docx]

***Supporting Material***

**5-methyl-2-phenyl-2,4-dihydro-3H-pyrazol-3-one** **(2a)**

Yellow solid; yield, 73%, mp, 130.7-131 ℃; ^1^H NMR (400 MHz, CDCl_3_, δ ppm): δ 7.85 (d, *J* =7.6 Hz, 2H, Ar-H), 7.38 (t, *J* =7.6 Hz, 2H, Ar-H), 7.17 (t, *J* =6.8 Hz, 1H, Ar-H), 3.43 (d, *J*=3.6 Hz, 2H, CH_2_), 2.18 (d, *J* =4 Hz, CH_3_). ^13^C NMR (100 MHz, CDCl_3_, δ ppm): δ 170.57, 156.33, 138.02, 128.83, 125.04, 118.87, 43.12, 17.05. HRMS (ESI) C_10_H_10_N_2_O [M+H]^+^: calcd.175.0866, found 175.0862.

**5-methyl-2-(o-tolyl)-2,4-dihydro-3H-pyrazol-3-one** (**2b**)

White solid; yield, 53%, mp, 168.2-169.9 ℃; ^1^H NMR (400 MHz, CDCl_3_, δ ppm): δ 7.28-7.38 (m, 6.37 H, Ar-H), 5.79 (s, 1H, CH_2_), 2.55 (s, 4.28 H, Ar-CH_3_), 2.52 (s, 3.25 H, Ar-CH_3_), 2.19 (s, 3.53 H, N-C-CH_3_). ^13^C NMR (100 MHz, CDCl_3_, δ ppm): δ: 160.11, 153.43, 151.20, 144.08, 135.14, 134.80, 131.25, 129.66, 127.16, 126.77, 105.06, 100.98, 19.77, 17.82, 14.76. HRMS (ESI) C_11_H_12_N_2_O [M+H]^+^: calcd.189.1022, found 189.1018.

**5-methyl-2-(m-tolyl)-2,4-dihydro-3H-pyrazol-3-one** (**2c**)

Light yellow; yield, 80%; solid, mp, 109.5-110.1 ℃; ^1^H NMR (400 MHz, CDCl_3_, δ ppm): δ 7.65 (d, *J* = 7.2 Hz, 2H, Ar-H), 7.28 (t, *J* = 7.6 Hz, 1H, Ar-H), 6.99 (d, *J* = 6.9 Hz, 1H, Ar-H), 3.41 (s, 2H, CH_2_), 2.38 (s, 3H, Ar-CH_3_), 2.19 (s, 3H, N-C-CH_3_). ^13^C NMR (100 MHz, CDCl_3_, δ ppm): δ 170.55, 156.24, 138.76, 137.93, 128.67, 125.91, 119.51, 116.13, 43.13, 21.61, 17.05. HRMS (ESI) C_11_H_12_N_2_O [M+H]^+^: calcd.189.1022, found 189.1018.

**5-methyl-2-(p-tolyl)-2,4-dihydro-3H-pyrazol-3-one** (**2d**)

White solid; yield, 74%, mp, 132.3-132.6 ℃; ^1^H NMR (400 MHz, CDCl_3_, δ ppm): δ 7.71 (d, *J* = 8.4, 2H, Ar-H), 7.18 (d, *J* = 8.4 Hz, 2H, Ar-H), 3.40 (s, 2H, CH_2_), 2.34 (s, 3H, Ar-CH_3_), 2.17 (s, 3H, N-C-CH_3_). ^13^C NMR (100 MHz, CDCl_3_, δ ppm): δ 170.45, 156.21, 135.60, 134.73, 129.34, 118.97, 43.06, 20.97, 17.02. HRMS (ESI) C_11_H_12_N_2_O [M+H]^+^: calcd. 189.1022, found 189.1018.

**2-(2,4-dimethylphenyl)-5-methyl-2,4-dihydro-3H-pyrazol-3-one** (**2e**)

White solid; yield, 60%, mp, 158.4-158.7 ℃; ^1^H NMR (400 MHz, CDCl_3_, δ ppm): δ 7.59 (s, 1H, Ar-H), 7.55 (d, *J* = 8.0 Hz, 1H, Ar-H), 7.13 (d, *J* = 8.0 Hz, 1H, Ar-H), 3.39 (s, 2H, CH_2_), 2.28 (s, 3H, Ar-CH_3_), 2.24 (s, 3H, Ar-CH_3_), 2.17 (s, 3H, N-C-CH_3_). ^13^C NMR (100 MHz, CDCl_3_, δ ppm): δ 170.42, 156.07, 137.07, 135.85, 133.49, 129.81, 120.24, 116.63, 43.03, 19.96, 19.26, 16.97. HRMS (ESI) C_12_H_14_N_2_O [M+H]^+^: calcd.203.1179, found 203.118.

**2-(3,4-dimethylphenyl)-5-methyl-2,4-dihydro-3H-pyrazol-3-one** (**2f**)

Light brown solid; yield, 62%, mp, 121-122 ℃; ^1^H NMR (400 MHz, CDCl_3_, δ ppm): δ 7.60 (d, *J* = 2.0 Hz, 1H, Ar-H), 7.53-7.56 (m, 1H, Ar-H), 7.13 (d, *J* = 8.4 Hz, 1H, Ar-H), 3.40 (s, 2H, CH_2_), 2.28 (s, 3H, Ar-CH_3_), 2.24 (s, 3H, Ar-CH_3_), 2.18 (s, 3H, N-C-CH_3_). ^13^C NMR (100 MHz, CDCl_3_, δ ppm): δ 170.41, 156.04, 137.10, 135.81, 133.54, 129.82, 120.28, 116.66, 43.05, 19.97, 19.27, 17.01. HRMS (ESI) C_12_H_14_N_2_O [M+H]^+^: calcd.203.1179, found 203.1175.

**2-(2-chlorophenyl)-5-methyl-2,4-dihydro-3H-pyrazol-3-one** (**2g**)

Orange solid; yield, 41%, mp, 105.1-105.9 ℃; ^1^H NMR (400 MHz, CDCl_3_, δ ppm) δ 7.48-7.51 (m, 1H, Ar-H), 7.40-7.42 (m, 1H, Ar-H), 7.30-7.36 (m, 2H, Ar-H), 3.41 (s, 2H, CH_2_), 2.18 (s, 3H, N-C-CH_3_). ^13^C NMR (100 MHz, CDCl_3_, δ ppm): δ 171.23, 156.52, 134.49, 131.84, 130.54, 129.81, 128.85, 127.53, 41.44, 17.10. HRMS (ESI) C_10_H_9_N_2_OCl [M+H]^+^: calcd.209.0476, found 209.0478.

**2-(3-chlorophenyl)-5-methyl-2,4-dihydro-3H-pyrazol-3-one** (**2h**)

Light yellow solid; yield, 54%, mp, 154-154.3 ℃; ^1^H NMR (400 MHz, CDCl_3_, δ ppm): δ 7.91 (t, *J* = 1.2 Hz, 1H, Ar-H), 7.81 (d, *J* = 8.0 Hz, 2H, Ar-H), 7.40 (t, *J* = 8.0 Hz, 1H, Ar-H), 7.29 (d, J = 8.0 Hz, 1H, Ar-H), 5.84 (s, 1H, CH), 2.52 (s, 3H, N-C-CH_3_). ^13^C NMR (100 MHz, CDCl_3_, δ ppm): δ 159.41, 153.16, 150.42, 144.78, 137.95, 135.20, 130.39, 127.10, 120.66, 118.27, 105.58, 102.57, 19.78, 14.76. HRMS (ESI) C_10_H_9_N_2_OCl [M+H]^+^: calcd.209.0476, found 209.0479.

**2-(4-chlorophenyl)-5-methyl-2,4-dihydro-3H-pyrazol-3-one** (**2i**)

White solid; yield, 75%, mp, 148-151.3 ℃; ^1^H NMR (400 MHz, CDCl_3_, δ ppm): δ 9.68 (s, 1H), 7.89 (s, 1H, Ar-H), 7.16 (d, *J* = 8.8 Hz, 2H, Ar-H), 6.69 (d, *J* = 8.8 Hz, 2H, Ar-H), 1.89 (s, 3H, N-C-CH_3_). ^13^C NMR (100 MHz, CDCl_3_, δ ppm): δ 169.54, 148.82, 128.90, 122.05, 113.97, 113.63, 21.05. HRMS (ESI) C_11_H_11_N_2_OCl [M+H]^+^: HRMS (ESI) C_10_H_9_N_2_OCl [M+H]^+^: calcd.209.0476, found 209.0478.

**2-(2,4-dichlorophenyl)-5-methyl-2,4-dihydro-3H-pyrazol-3-one** (**2j**)

White solid; yield, 70%, mp, 155.9-156.2 ℃; ^1^H NMR (400 MHz, DMSO, δ ppm): δ 9.80 (s, 1H), 7.57 (s, 1H, , Ar-H), 7.40 (d, *J* = 2.0, 1H, Ar-H), 7.15-7.18 (m, 1H, Ar-H), 7.20-7.22 (m, 1 H, Ar-H), 6.75 (d, *J* = 8.8 Hz, 1H, CH), 1.92 (s, 3H, N-C-CH_3_). ^13^C NMR (100 MHz, DMSO, δ ppm): δ 169.56, 144.40, 128.11, 122.27, 117.93, 114.37, 21.04. HRMS (ESI) C_10_H_8_N_2_OCl_2_ [M+H]^+^: calcd.243.0086, found 243.0089.

**2-(3,4-dichlorophenyl)-5-methyl-2,4-dihydro-3H-pyrazol-3-one** (**2k**)

White solid; yield, 71%, mp, 182-182.8 ℃; ^1^H NMR (400 MHz, CDCl_3_, δ ppm): δ 8.02 (d, *J* = 2.8 Hz, 1H, Ar-H), 7.77-7.80 (m, 1H, Ar-H), 7.52 (d, *J* = 8.8 Hz, 1H, Ar-H), 5.84 (s, 1H, CH), 2.43 (s, 3H). ^13^C NMR (100 MHz, CDCl_3_, δ ppm): δ 159.19, 153.19, 150.05, 145.05, 136.21, 133.45, 130.93, 130.65, 121.83, 118.98, 105.61, 102.66, 19.79, 14.75. C_10_H_8_N_2_OCl_2_ [M+H]^+^: calcd.243.0086, found 243.0085.

**2-(2-fluorophenyl)-5-methyl-2,4-dihydro-3H-pyrazol-3-one** (**2l**)

White solid; yield, 75%, mp, 135.3-138.1 ℃; ^1^H NMR (400 MHz, CDCl_3_, δ ppm): δ 7.42-7.46 (m, 1H, Ar-H), 7.28-7.34 (m, 1H, Ar-H), 7.16-7.21 (m, 2H, Ar-H), 3.39 (s, 2H, CH_2_), 2.18 (s, 3H, N-C-CH_3_). ^13^C NMR (100 MHz, CDCl_3_, δ ppm): δ 171.05, 157.79, 156.90, 155.28, 129.37, 129.30, 127.07, 127.06, 124.74, 124.62, 124.40, 124.37, 116.88, 116.69, 41.45, 17.03. HRMS (ESI) C_11_H_12_N_2_O [M-H]^-^: calcd.191.0626, found 191.0624.

**2-(3-fluorophenyl)-5-methyl-2,4-dihydro-3H-pyrazol-3-one** (**2m**)

Gray solid; yield, 65%, mp, 121.8-122 ℃; ^1^H NMR (400 MHz, CDCl_3_, δ ppm): δ 7.48-7.51 (m, 1H, Ar-H), 7.39-7.42 (m, 1H, Ar-H), 7.30-7.36 (m, 2H, Ar-H), 3.41 (s, 2H, CH_2_), 2.18 (s, 3H, N-C-CH_3_). ^13^C NMR (100 MHz, CDCl_3_, δ ppm): δ 170.55, 164.04, 156.63, 139.53, 139.42,131.61, 130.10, 130.01, 113.87, 113.84, 111.58, 111.37,16.93, 106.06, 105.79, 43.13. HRMS (ESI) C_11_H_12_N_2_O [M-H]^-^: calcd.191.0626, found 191.0625.

**2-(4-fluorophenyl)-5-methyl-2,4-dihydro-3H-pyrazol-3-one** (**2n**)

Gray yellow solid; yield, 66%, mp, 151.9-152.8 ℃, ^1^H NMR (400 MHz, CDCl_3_, δ ppm): δ 7.81-7.84 (m, 2H, Ar-H), 7.05-7.09 (m, 2H, Ar-H), 3.42 (s, 2H, CH_2_), 2.18 (s, 3H, N-C-CH_3_). ^13^C NMR (100 MHz, CDCl_3_, δ ppm): δ 170.38, 160.98, 158.55, 156.48, 134.25, 134.23, 120.57, 120.49, 115.55, 115.32, 42.94, 16.92. HRMS (ESI) C_11_H_12_N_2_O [M-H]^-^: calcd.191.0626, found 191.0626.

**2-benzyl-3-methyl-1H-pyrazol-5-ol** (**5a**)

White solid; yields, 71%, mp 160.3-160.8 ℃; ^1^H NMR (400 MHz, CDCl_3_, δ ppm): δ 7.30 (m, 2H, Ar-H), 7.23-7.27 (m, 1H, Ar-H), 7.13 (d, *J* =8.4 Hz, 2H, Ar-H), 5.43 (s, 1H, CH), 5.06 (s, 2H, Ar-CH_2_), 2.13 (s, 3H, N-C-CH_3_). ^13^C NMR (100 MHz, CDCl_3_, δ ppm): δ 161.55, 140.47, 136.87, 128.75, 127.58, 126.73, 91.47, 51.93, 11.38. HRMS (ESI) C_11_H_12_N_2_O [M-H]^-^: calcd.187.0877, found 187.0874.

**5-methyl-2-(2-methylbenzyl)-2,4-dihydro-3H-pyrazol-3-one** (**5b**)

White solid, yield, 57%; mp, 173.8-176 ℃; ^1^H NMR (400 MHz, DMSO, δ ppm): δ 7.15-7.17 (m, 1.27H, Ar-H), 7.30 (t, *J* = 4.8 Hz, 1.4H, Ar-H), 7.04-7.06 (m, 2.59H, Ar-H), 6.95-6.97 (m, 1.4H, Ar-H), 5.36 (s, 0.65H, CH), 5.02 (s, 1H, Ar-CH_2_), 3.51 (s, 2.16H, CH_2_), 2.29 (s, 5H, Ar-CH_3_), 2.10 (d, 2H ,N-C-CH_3_), 1.93 (s, 3H, N-C-CH_3_). ^13^C NMR (100 MHz, DMSO, δ ppm): δ 160.47, 160.21, 139.99, 137.57, 136.86, 136.04, 135.52, 130.36, 130.07, 128.56, 127.41, 126.86, 126.32, 125.90, 98.99, 91.04, 49.70, 25.35, 19.65, 19.09, 11.34, 10.56. HRMS (ESI) C_12_H_14_N_2_O [M+H]^+^: calcd.203.1179, found 203.1178.

**5-methyl-2-(3-methylbenzyl)-2,4-dihydro-3H-pyrazol-3-one** (**5c**)

White solid; yield, 54%, mp, 204.8-205.3 ℃; ^1^H NMR (400 MHz, DMSO, δ ppm): δ 7.12 (t, *J* = 7.2 Hz, 1H, Ar-H), 6.93-6.96 (m, 3H, Ar-H), 3.50 (s, 2H, Ar-CH_2_), 3.34 (s, 2H, CH_2_), 2.25 (s, 3H, Ar-CH_3_), 2.00 (s, 3H, N-C-CH_3_). ^13^C NMR (100 MHz, DMSO, δ ppm): δ 142.27, 137.45, 128.41, 129.08, 126.50, 125.55, 100.46, 79.75, 79.42, 79.09, 27.70, 21.54, 10.47. HRMS (ESI) C_12_H_14_N_2_O [M+H]^+^: calcd.203.1179, found 203.1176.

**5-methyl-2-(4-methylbenzyl)-2,4-dihydro-3H-pyrazol-3-one** (**5d**)

White solid; yield, 52%, mp, 187.6-188.6 ℃; ^1^H NMR (400 MHz, CDCl_3_, δ ppm): δ 7.11 (d, *J* = 8.0 Hz, 2H, Ar-H), 7.03 (d, *J* = 8.0 Hz, 2H, Ar-H), 5.42 (s, 2H, Ar-CH_2_), 5.01 (s, 2H, CH_2_), 2.31 (s, 3H, Ar-CH_3_), 2.13 (s, 3H, N-C-CH_3_). ^13^C NMR (100 MHz, CDCl_3_, δ ppm): δ 161.51, 140.32, 137.22, 133.86, 129.40, 126.80, 91.40, 57.74, 21.10, 11.40. HRMS (ESI) C_12_H_14_N_2_O [M+H]^+^: calcd.203.1179, found 203.117.

**2-(2-chlorobenzyl)-5-methyl-2,4-dihydro-3H-pyrazol-3-one** (**5e**)

White solid; Yield, 41%, mp, 153.7-154.7 ℃; ^1^H NMR (400 MHz, DMSO, δ ppm): δ 7.39-7.41 (m, 1H, Ar-H), 7.38 (t, *J* = 7.8 Hz, 2H, Ar-H), 7.18-7.26 (m, 2H, Ar-H), 7.09-7.11 (m, 1H, CH), 3.64 (s, 2H, Ar-CH_2_), 1.99 (s, 3H, N-C-CH_3_). ^13^C NMR (100 MHz, DMSO, δ ppm): δ 138.89, 133.12, 130.47, 129.32, 127.89, 127.38, 98.11, 25.43, 10.48. HRMS (ESI) C_11_H_11_N_2_OCl [M+H]^+^: calcd.223.0633, found 223.0630.

**1-(3-chlorobenzyl)-3-methyl-1H-pyrazol-5-ol (5f)**

Light yellow solid; yield, 34%, mp, 220.6-223.5 ℃; ^1^H NMR (400 MHz, DMSO, δ ppm): δ 7.27 (t, *J* = 8.0 Hz, 1H, Ar-H), 7.21 (s, 1H, Ar-H), 7.18 (s, 2H, Ar-H), 7.14 (d, *J* = 7.6 Hz, 1H, CCH), 3.57 (s, 2H, Ar-CH_2_), 2.02 (s, 3H, N-C-CH_3_). ^13^C NMR (100 MHz, DMSO, δ ppm): δ 160.04, 145.02, 133.23, 130.42, 128.17, 127.18, 125.91, 99.74, 27.39, 10.36. HRMS (ESI) C_11_H_11_N_2_OCl [M+H]^+^: calcd.223.0633, found 223.0630.

**2-(4-chlorobenzyl)-5-methyl-2,4-dihydro-3H-pyrazol-3-one** (**5g**)

Light yellow solid; yield, 42%, mp, 132.1-132.4 ℃; ^1^H NMR (400 MHz, CDCl_3_, δ ppm): δ 7.31 (s, 1H, Ar-H), 7.24-7.26 (m, 2H, Ar-H), 7.19-7.21 (m, 1H, Ar-H), 4.76 (s, 2H, Ar-CH_2_), 3.23 (s, 2H, CH_2_), 2.07 (s, 3H, N-C-CH_3_). ^13^C NMR (100 MHz, CDCl_3_, δ ppm): δ 172.05, 156.04, 138.54, 134.47, 129.92, 128.19, 127.92, 126.30, 47.19, 41.48, 17.03. HRMS (ESI) C_11_H_11_N_2_OCl [M+H]^+^: calcd.223.0633, found 223.0630.

**5-methyl-2-(2-nitrobenzyl)-2,4-dihydro-3H-pyrazol-3-one (5h)**

Light yellow solid; yield, 38%, mp, 179.2-180.5 ℃; ^1^H NMR (400 MHz, DMSO, δ ppm): δ 8.01 (d, *J* = 8.0, 1H, Ar-H), 7.92 (d, *J* = 8.4, 2H, Ar-H), 7.64 (t, *J* = 8.4, 2H, Ar-H), 7.56 (t, *J* = 8.0, 2H, Ar-H), 7.49 (t, *J* = 7.6, 1H, Ar-H), 7.42 (t, *J* = 7.6, 1H, Ar-H), 7.35 (d, *J* = 7.6, 2H, Ar-H), 6.03 (d, *J* = 8.0, 1H), 4.67 (s, 2H, Ar-CH_2_), 2.15 (s, 3H, N-C-CH_3_). ^13^C NMR (100 MHz, DMSO, δ ppm): δ 174.91, 161.68, 150.15, 148.00, 134.24, 133.46, 133.08, 131.42, 129.36, 129.28, 129.00, 128.58, 125.37, 125.33, 58.61, 44.07, 34.63, 14.22. HRMS (ESI) C_11_H_11_N_3_O_3_ [M+H]^+^: calcd.234.0873, found 234.0867.

**3-methyl-1-(3-nitrobenzyl)-1H-pyrazol-5-ol** (**5i**)

Light yellow solid; yield, 42%, mp, 202.9-203 ℃; ^1^H NMR (400 MHz, DMSO, δ ppm): δ 9.59 (s, 1H, OH), 8.13 (d, *J* = 8.0 Hz, 1H, Ar-H), 7.94 (s, 1H, Ar-H), 7.64 (t, *J* = 8.0, 1H, Ar-H), 7.56 (d, *J* = 7.6 Hz, 1H, Ar-H), 5.38 (s, 1H, CH), 5.20 (s, 2H, Ar-CH_2_), 2.16 (s, 3H, N-C-CH_3_). ^13^C NMR (100 MHz, DMSO, δ ppm): δ 160.94, 148.33, 141.05, 140.17, 134.06, 130.50, 122.64, 121.96, 91.60, 50.61, 11.28. HRMS (ESI) C_11_H_11_N_3_O_3_ [M+H]^+^: calcd.234.0873, found 234.0867.

**1-ethyl-3-methyl-1H-pyrazol-5-ol** (**5j**)

Yellow solid, 40%; yield, mp, 149.7-151.1 ℃; ^1^H NMR (400 MHz, CDCl_3_, δ ppm): δ 5.37 (s, 1H, CH), 3.84-3.90 (m, 2H, CH_2_), 2.17 (s, 3H, N-C-CH_3_), 1.34 (t, *J* = 7.2 Hz, 3H, CH_2_-CH_3_). ^13^C NMR (100 MHz, CDCl_3_, δ ppm): δ 161.41, 139. 37, 90.38, 42.73, 15.35, 11.10. HRMS (ESI) C_6_H_10_N_2_O [M+H]^+^: calcd.127.0866, found 127.0866

**3-methyl-1-phenyl-1H-pyrazol-5-yl 3,7-dichloroquinoline-8-carboxylate** (**8a**)*.* Yield, 62%; light yellow solid, mp,135.7-135.9 ℃; ^1^H NMR (400 MHz, CDCl_3_, δ ppm): δ 8.80 (d, *J* =1.6 Hz, 1H, Quinoline-H), 8.16 (d, *J* =2.0 Hz,1H, Quinoline-H), 7.80 (d, *J* =8.8 Hz, 1H, Quinoline-H), 7.63-7.65 (m, 2H, Ar-H), 7.59 (d, *J* =8.8 Hz, 1H, Quinoline-H), 7.32-7.36 (m, 2H, Ar-H), 7.26-7.28 (m, 1H, Ar-H), 6.47 (s, 1H, CH), 2.40 (s, 3H, N-C-CH_3_). ^13^C NMR (100 MHz, CDCl_3_, δ ppm): δ161.28, 151.24, 149.04, 144.08, 143.60, 137.91, 133.84, 132.48, 131.09, 129.83, 128.82, 127.06, 126.57, 123.57, 96.11, 14.58. HRMS (ESI) C_20_H_13_N_3_O_2_Cl_2_ [M+H]^+^: calcd. 398.0458, found 398.0459.

**3-methyl-1-(o-tolyl)-1H-pyrazol-5-yl 3,7-dichloroquinoline-8-carboxylate** (**8b**)

White solid; yield, 53%, mp, 161.6-162.6 ℃; ^1^H NMR (400 MHz, DMSO, δ ppm): δ 8.90 (d, *J* = 2.4 Hz, 1H, Quinoline-H), 8.69 (d, *J* = 2.4 Hz, 1H, Quinoline-H), 8.13 (d, *J* = 8.8 Hz, 1H, Quinoline-H), 7.78 (d, *J* = 8.8 Hz, 1H, Quinoline-H), 7.34-7.37 (m, 2H, Ar-H), 7.28-7.29 (m, 2H, Ar-H), 6.36 (s, 1H, CH), 2.30 (s, 3H, Ar-CH_3_), 2.08 (s, 3H, N-C-CH_3_). ^13^C NMR (100 MHz, DMSO, δ ppm): δ 161.69, 151.80, 148.34, 144.53, 143.14, 136.55, 136.07, 135.30, 131.68, 131.68, 131.15, 130.18, 129.74, 129.45, 129.03, 127.05, 126.90, 95.11, 17.45, 14.70. HRMS (ESI) C_20_H_11_N_3_O_2_Cl_4_ [M+H]^+^: calcd. 412.0614, found 412.0616.

**3-methyl-1-(m-tolyl)-1H-pyrazol-5-yl 3,7-dichloroquinoline-8-carboxylate (8c)**

Light yellow solid; yield, 56%, mp, 110.3-111.1 ℃; ^1^H NMR (400 MHz, DMSO, δ ppm): δ 9.01 (d, *J* = 2.4 Hz, 1H, Quinoline-H), 8.76 (d, *J* = 2.4 Hz, 1H, Quinoline-H), 8.22 (d, *J* = 9.2 Hz, 1H, Quinoline-H), 7.89 (d, *J* = 8.8 Hz, 1H, Quinoline-H), 7.38 (d, *J* = 9.2 Hz, 2H, Ar-H), 7.31 (t, *J* = 8.8 Hz, 1H, Ar-H), 7.12 (d, *J* = 8.4 Hz, 1H, Ar-H), 6.43 (s, 1H, CH), 2.30 (s, 3H, Ar-CH_3_), 2.22 (s, 3H, N-C-CH_3_). ^13^C NMR (100 MHz, DMSO, δ ppm): δ 161.63, 151.97, 148.67, 143.79, 143.20, 139.02, 137.72, 135.38, 130.19, 131.96, 131.87, 129.54, 129.35, 129.11, 128.29, 127.13, 123.63, 120.38, 96.49, 21.20, 14.63,. HRMS (ESI) C_21_H_15_N_3_O_2_Cl_2_ [M+H]^+^: calcd. 412.0614, found 412.0616.

**3-methyl-1-(p-tolyl)-1H-pyrazol-5-yl 3,7-dichloroquinoline-8-carboxylate** (**8d**)

Brownness solid; yield, 53%, mp, 125.1-126.4 ℃; ^1^H NMR (400 MHz, DMSO, δ ppm): δ 9.01 (d, *J* = 2.4 Hz,1H, Quinoline-H), 8.78 (d, *J* = 2.4 Hz, 1H, Quinoline-H), 8.23 (d, *J* = 9.2 Hz, 1H, Quinoline-H), 7.90 (d, *J* = 8.8 Hz, 1H, Quinoline-H), 7.46 (d, *J* = 8.4 Hz, 2H), 7.23 (d, *J* = 8.0 Hz, 2H), 6.41 (s, 1H, CH), 2.31 (s, 3H), 2.29 (s, 3H, N-C-CH_3_). ^13^C NMR (100 MHz, DMSO, δ ppm): δ 161.78, 151.98, 148.52, 143.71, 143.20, 137.28, 135.44, 135.38, 132.00, 131.87, 130.34, 130.21, 129.97, 129.58, 129.15, 127.18, 123.28, 121.10, 96.39, 20.95, 14.64. HRMS (ESI) C_22_H_17_N_3_O_2_Cl_2_ [M+H]^+^: calcd. 412.0614, found 412.0607.

**1-(2,4-dimethylphenyl)-3-methyl-1H-pyrazol-5-yl 3,7-dichloroquinoline-8-carboxylate (8e)**

White solid; yield, 68%, mp, 251.8-252.2 ℃; ^1^H NMR (400 MHz, DMSO, δ ppm): δ 8.89 (d, *J* = 2.4 Hz, 1H, Quinoline-H), 8.70 (d, *J* = 2.4 Hz, 1H, Quinoline-H), 8.14 (d, *J* = 8.8 Hz, 1H, Quinoline-H), 7.80 (d, *J* = 8.8 Hz, 1H, Quinoline-H), 7.15 (d, *J* = 7.6 Hz, 2H, Ar-H), 7.06 (d, *J* = 9.4 Hz, 1H, Ar-H), 6.35 (s, 1H, CH), 2.29 (s, 6H, Ar-CH_3_), 2.03 (s, 3H, N-C-CH_3_). ^13^C NMR (100 MHz, DMSO, δ ppm): δ 161.59, 151.79, 144.58, 143.14, 139.22, 135.69, 135.29, 134.12, 131.81, 131.67, 131.59, 130.24, 129.44, 129.05, 128.10, 127.35, 127.06, 94.91, 21.06, 17.36,14.70. HRMS (ESI) C_22_H_17_N_3_O_2_Cl_2_ [M+H]^+^: calcd. 426.0771, found 426.0773.

**1-(3,4-dimethylphenyl)-3-methyl-1H-pyrazol-5-yl 3,7-dichloroquinoline-8-carboxylate (8f).**

White solid; yield, 58%, mp, 215.4-215.8 ℃; ^1^H NMR (400 MHz, CDCl_3_, δ ppm): δ 8.79 (d, *J* = 2.4 Hz, 1H, Quinoline-H), 8.14 (d, *J* = 2.4 Hz, 1H, Quinoline-H), 7.78 (d, *J* = 8.8 Hz, 1H, Quinoline-H), 7.57 (d, *J* = 8.8 Hz, 1H, Quinoline-H), 7.38 (s, 1H, Ar-H), 7.33 (d, *J* = 8.0 Hz, 1H, Ar-H), 7.06 (d, *J* = 8.0 Hz, 1H, Ar-H), 6.46 (s, 1H, CH), 2.39 (s, 3H, Ar-CH_3_), 2.22 (s, 3H, Ar-CH_3_), 2.18 (s, 3H, N-C-CH_3_). ^13^C NMR (100 MHz, CDCl_3_, δ ppm): δ 161.21, 151.24, 148.64, 144.00, 143.64, 137.23, 136.67, 135.65, 133.79, 132.44, 131.21, 129.78, 128.81, 126.55, 124.68, 120.95, 95.69, 19.70, 19.34, 14.57. HRMS (ESI) C_22_H_17_N_3_O_2_Cl_2_ [M+H]^+^: calcd. 426.0771, found 426.0773.

**1-(2-chlorophenyl)-3-methyl-1H-pyrazol-5-yl 3,7-dichloroquinoline-8-carboxylate (8g)**

Light yellow solid; yield, 51%, mp, 118.1-118.5 ℃; ^1^H NMR (400 MHz, CDCl_3_, δ ppm): δ 9.78 (s, 1H, Quinoline-H), 7.35 (s, 1H, Quinoline-H ), 7.28 (d, 1H, Quinoline-H), 7.15 (d, *J* = 8.8 Hz, 1H, Quinoline-H), 6.72-6.81 (m, 4H, Ar-CH), 6.42 (s, 1H, CH), 1.93 (s, 3H, N-C-CH_3_). ^13^C NMR (100 MHz, CDCl_3_, δ ppm): δ 176.91, 170.25, 143.69, 142.83, 129.64, 129.47, 128.07, 127.62, 121.43, 121.31, 119.42, 118.39, 113.46, 112.53, 20.81, 19.12. HRMS (ESI) C_20_H_12_N_3_O_2_Cl_3_ [M+H]^+^: calcd. 432.0068, found 432.0070.

**1-(3-chlorophenyl)-3-methyl-1H-pyrazol-5-yl 3,7-dichloroquinoline-8-carboxylate (8h)**

White solid; yield, 61%, mp, 262.9-263.0 ℃; ^1^H NMR (400 MHz, DMSO, δ ppm): δ 11.12 (s, 1H, OH ), 9.03 (d, *J* = 2.4 Hz, 1H, Quinoline-H), 8.72 (s, 1H, Ar-CH), 8.62 (d, *J* = 2.4 Hz, 1H, Quinoline-H), 8.08 (d, *J* = 8.8 Hz, 1H, Quinoline-H), 7.97 (d, *J* = 9.2 Hz, 1H, Quinoline-H), 7.80 (s, 1H, Ar-CH), 7.77 (t, *J* = 2.0 Hz, 2H, Quinoline-H), 7.66 (d, *J* = 8.8 Hz, 1H, Quinoline-H), 7.59-7.61 (m, 1H, Ar-CH), 7.51 (t, *J* = 8.0 Hz, 1H, Quinoline-H), 7.37-7.39 (m, 1H, Ar-CH), 7.32 (t, *J* = 1.6 Hz, 1H, Ar-CH), 7.13-7.17 (m, 2H, Ar-CH), 7.11 (t, *J* = 1.6 Hz, 1H, CH), 7.08 (d, *J* = 8.0 Hz, 1H, CH), 1.97 (s, 3H, N-C-CH_3_), 1.42 (s, 3H, N-C-CH_3_). ^13^C NMR (100 MHz, DMSO, δ ppm): δ 168.77, 164.06, 150.96, 143.27, 142.86, 142.70, 135.25, 133.85, 133.34, 132.56, 131.94, 130.95, 130.68, 130.46, 129.99, 129.22, 128.95, 128.77, 128.49, 126.86, 126.69, 126.49, 125.44, 21.02, 20.45. HRMS (ESI) C_20_H_12_N_3_O_2_Cl_3_ [M-H]^-^: calcd. 429.9922, found 429.9932.

**1-(4-chlorophenyl)-3-methyl-1H-pyrazol-5-yl 3,7-dichloroquinoline-8-carboxylate** (**8i**)

White solid; yield, 58% , mp, 235.4-235.9 ℃; ^1^H NMR (400 MHz, DMSO, δ ppm): δ 11.10 (s, 1H ), 9.00 (d, *J* = 2.4 Hz, 1H, Quinoline-H), 8.72 (s, 0.72H), 8.61 (d, *J* = 2.4 Hz, 1H, Quinoline-H), 8.07 (d, *J* = 9.2 Hz, 0.79H, Quinoline-H), 7.96 (d, *J* = 8.8 Hz, 1H, Quinoline-H), 7.78 (d, *J* = 8.8 Hz, 1H, Quinoline-H), 7.70 (t, *J* = 3.2 Hz, 0.71H, Ar-CH), 7.67 (t, *J* = 2.0 Hz,1H, Quinoline-H), 7.64 (d, *J* = 8.8 Hz, 1H, Quinoline-H), 7.55 (t, *J* = 3.2 Hz, 1H, Ar-CH), 7.53 (t, *J* = 2.0 Hz, 0.70H, Quinoline-H), 7.27 (t, *J* = 3.2 Hz, 1H, Ar-CH), 7.24 (t, *J* = 2.4 Hz, 1.47H, Ar-CH), 7.15 (t, *J* = 3.2 Hz, 1.42H, Ar-CH), 7.13 (t, *J* = 2.0 Hz, 1H, CH), 1.96 (s, 3H, N-C-CH_3_), 1.41 (s, 2.2H, N-C-CH_3_). ^13^C NMR (100 MHz, DMSO, δ ppm): δ 168.64, 164.07, 151.01, 143.28, 140.58, 140.34, 135.24, 133.98, 132.80, 131.84, 130.59, 129.91, 129.22, 129.14, 128.90, 128.74, 128.56, 126.87, 21.02, 20.45. HRMS (ESI) C_20_H_12_N_3_O_2_Cl_3_ [M-H]^-^: calcd. 429.9922, found 429.9933.

**1-(2,4-dichlorophenyl)-3-methyl-1H-pyrazol-5-yl 3,7-dichloroquinoline-8-carboxylate (8j)**

white solid; yield, 68%, mp, 184.9-186.9 ℃; ^1^H NMR (400 MHz, DMSO, δ ppm): δ 8.85 (d, *J* = 2.4 Hz, 1H, Quinoline-H), 8.74 (d, *J* = 2.4 Hz, 1H, Quinoline-H), 8.17-8.20 (m, 1H, Ar-H), 7.86 (t, *J* = 8.8 Hz, 2H, Quinoline-H), 7.59 (d, *J* = 2.4 Hz, 2H, Ar-H), 6.40 (s, 1H, CH), 2.30 (s, 3H, N-C-CH_3_). ^13^C NMR (100 MHz, DMSO, δ ppm): δ 161.45, 151.73, 149.65, 145.00, 143.14, 135.39, 135.36, 135.36, 134.13, 132.97, 131.98, 131.91, 131.71, 130.10, 129.96, 129.48, 129.09, 128.71, 127.10, 95.85, 14.69. HRMS (ESI) C_20_H_11_N_3_O_2_Cl_4_ [M+H]^+^: calcd. 465.9678, found 465.9679.

**1-(3,4-dichlorophenyl)-3-methyl-1H-pyrazol-5-yl 3,7-dichloroquinoline-8-carboxylate** (**8k**)

Light yellow solid; yield, 75%, mp, 164.5-165.5℃; ^1^H NMR (400 MHz, DMSO, δ ppm): δ 11.17 (s, 0.87H), 9.04 (d, *J* = 2.4 Hz, 1.22H, Quinoline-H), 8.72 (s, 1.06H, Ar-CH), 8.64 (d, *J* = 2.4 Hz, 1.07H, Quinoline-H), 8.09 (d, *J* = 9.2 Hz, 1.07H, Quinoline-H), 7.99 (d, *J* = 8.8 Hz, 1.07H, Quinoline-H), 7.95 (d, *J* = 2.4 Hz, 0.97H, Quinoline-H), 7.80 (s, 0.88H, Ar-CH), 7.76 (d, *J* = 8.8 Hz, 1.15H, Quinoline-H), 7.68 (d, *J* = 8.8 Hz, 0.97H, Quinoline-H), 7.62-7.65 (m, 1.11H, Ar-CH), 7.49 (d, *J* = 2.8 Hz, 1.01H, Ar-CH), 7.35 (d, *J* = 8.4 Hz, 0.93H, Ar-CH), 7.15-7.18 (m, 0.99H, Ar-CH), 1.98 (s, 2.56H, N-C-CH_3_), 1.43 (s, 3H, N-C-CH_3_),. ^13^C NMR (100 MHz, DMSO, δ ppm): δ 168.90, 163.95, 151.13, 143.24, 141.39, 141.21, 135.34, 133.55, 131.95, 131.48, 131.21, 131.05, 130.91, 130.89, 130.84, 130.10, 129.24, 129.06, 128.82, 128.53, 127.00,126.91, 21.00, 20.45. HRMS (ESI) C_20_H_11_N_3_O_2_Cl_4_ [M+H]^+^: calcd. 465.9678, found 465.9676.

**1-(2-fluorophenyl)-3-methyl-1H-pyrazol-5-yl 3,7-dichloroquinoline-8-carboxylate (8l)**

White solid; yield, 67%, mp, 217.3-217.6 ℃; ^1^H NMR (400 MHz, CDCl_3_, δ ppm): δ 8.70 (d, *J* = 2.4 Hz, 1H, Quinoline-H), 8.11 (d, *J* = 2.4 Hz, 1H, Quinoline-H), 7.76 (d, *J* = 9.2 Hz, 1H, Quinoline-H), 7.53-7.57 (m, 2H, Quinoline-H, Ar-H), 7.32-7.37 (m, 1H, Ar-H), 7.20 (t, *J* = 7.6 Hz, 1H, Ar-H), 7.10-7.15 (m, 1H, Ar-H), 6.43 (s, 1H, CH), 2.40 (s, 3H, N-C-CH_3_). ^13^C NMR (100 MHz, CDCl_3_, δ ppm): δ 161.22, 158.20, 155.68, 151.17, 150.04, 145.31, 143.65, 133.69, 132.45, 130.96, 130.15, 130.07, 129.72, 129.04, 128.77, 126.48, 125.72, 125.60, 124.35, 124.31, 116.52, 116.32, 95.52, 14.63. HRMS (ESI) C_20_H_12_N_3_O_2_Cl_2_F [M+H]^+^: calcd. 416.0363, found 416.0360.

**1-(3-fluorophenyl)-3-methyl-1H-pyrazol-5-yl 3,7-dichloroquinoline-8-carboxylate (8m)**

White solid; yield, 61%, mp, 230.3-231.2 ℃, ^1^H NMR (400 MHz, DMSO, δ ppm): δ 11.11 (s, 1H), 9.02 (d, *J* = 2.4 Hz, 1H, Quinoline-H), 8.99 (d, *J* = 2.4 Hz, 0.91H, Quinoline-H), 8.71 (d, *J* = 2.4 Hz, 1H, Quinoline-H), 8.61 (d, *J* = 2.4 Hz, 0.86H, Quinoline-H), 8.08-8.09 (m, 1.58H, Ar-CH), 7.96 (d, *J* = 8.8, 0.94H, Quinoline-H), 7.80 (d, *J* = 9.2 Hz, 1H, Quinoline-H), 7.65 (d, *J* = 8.8 Hz, 0.98H, Quinoline-H), 7.52-7.56 (m, 1H, Ar-CH), 7.50-7.51 (m, 1H, Ar-CH), 7.08-7.17 (m, 2.72H, Ar-CH), 7.05-7.06 (m, 0.91H, CH), 6.91 (t, *J* = 8.0 Hz, 1H, CH), 1.97 (s, 3H, N-C-CH_3_), 1.43 (s, 3H, N-C-CH_3_). ^13^C NMR (100 MHz, DMSO, δ ppm): δ 168.75, 166.90, 164.09, 163.53, 151.22, 150.99, 143.39, 143.32, 143.21, 143.17, 143.07, 143.03, 142.93, 135.26, 135.06, 133.96, 131.94, 130.92, 130.83, 130.64, 130.49, 130.40, 130.29, 129.95, 129.87, 129.23, 129.17, 128.98, 128.95, 128.77, 127.12, 126.87, 122.98, 122.95, 115.62, 115.41, 113.82, 113.59, 21.02, 20.44. HRMS (ESI) C_20_H_12_N_3_O_2_Cl_2_F [M+H]^+^: calcd. 416.0353, found 416.0363.

**1-(4-fluorophenyl)-3-methyl-1H-pyrazol-5-yl 3,7-dichloroquinoline-8-carboxylate (8n)**

Light yellow solid; yield, 67%, mp, 185-185.3 ℃; ^1^H NMR (400 MHz, DMSO, δ ppm): δ 9.03 (d, *J* = 2.4 Hz,1H, Quinoline-H), 8.78 (d, *J* = 2.4 Hz, 1H, Quinoline-H), 8.23 (d, *J* = 9.2 Hz, 1H, Quinoline-H), 7.90 (d, *J* = 8.8 Hz, 1H, Quinoline-H), 7.61-7.64 (m, 2H, Ar-H), 7.30-7.35 (m, 2H, Ar-H), 6.42 (s, 1H, CH), 2.30 (s, 3H, N-C-CH_3_). ^13^C NMR (100 MHz, DMSO, δ ppm): δ 162.55, 161.93, 152.05, 148.89, 143.79, 143.19, 135.44, 134.15, 132.05, 131.91, 130.07, 129.62, 129.14, 127.18, 125.70, 125.61, 116.58, 116.35, 96.70,14.62. HRMS (ESI) C_20_H_12_N_3_O_2_Cl_2_F [M+H]^+^: calcd. 416.0363, found 416.0356.

**1-benzyl-3-methyl-1H-pyrazol-5-yl 3,7-dichloroquinoline-8-carboxylate (9a)**

White solid; yield, 52%, mp, 193.5-195.2 ℃; ^1^H NMR (400 MHz, CDCl_3_, δ ppm): δ 8.88 (d, *J* = 2.4 Hz, 1H, Quinoline-H), 8.15 (d, *J* = 2.4 Hz, 1H, Quinoline-H), 7.79 (d, *J* = 8.8 Hz, 1H, Quinoline-H), 7.60 (d, *J* = 8.8 Hz, 1H, Quinoline-H), 7.23-7.33 (m, 3H, Ar-H), 7.15 (d, *J* = 8.8 Hz, 2H, Ar-H), 6.33 (s, 1H, CH), 5.24 (s, 2H, Ar-CH_2_), 2.24 (s, 3H, N-C-CH_3_). ^13^C NMR (100 MHz, CDCl_3_, δ ppm): δ 158.32, 149.18, 146.62, 139.03, 135.35, 131.82, 128.97, 127.62, 127.39, 124.83, 124.57, 124.09, 124.00, 122.95, 122.11, 121.81, 91.88, 48.30, 6.92. HRMS (ESI) C_21_H_15_N_3_O_2_Cl_2_ [M+H]^+^: calcd. 412.0614, found 412.0612.

**3-methyl-1-(2-methylbenzyl)-1H-pyrazol-5-yl 3,7-dichloroquinoline-8-carboxylate (9b)**

White solid; yield, 75%, mp, 193-194.7 ℃; ^1^H NMR (400 MHz, CDCl_3_, δ ppm): δ 8.87 (d, *J* = 2.4 Hz, 1H, Quinoline-H), 8.14 (d, *J* = 2.4 Hz, 1H, Quinoline-H), 7.79 (d, *J* = 8.8 Hz, 1H, Quinoline-H), 7.60 (d, *J* = 8.8 Hz, 1H, Quinoline-H), 7.11-7.17 (m, 3H, Ar-H), 6.68 (d, *J* = 7.6 Hz, 1H, Ar-H), 6.35 (s, 1H, CH), 5.23 (s, 2H, Ar-CH_2_), 2.34 (s, 3H, Ar-CH_3_), 2.22 (s, 3H, N-C-CH_3_). ^13^C NMR (100 MHz, CDCl_3_, δ ppm): δ 163.11, 153.94, 151.36, 143.77, 140.50, 134.96, 134.74, 133.71, 132.38, 130.24, 129.58, 129.31, 128.84, 127.55, 126.55, 126.45, 96.57, 51.03, 19.17, 11.65. HRMS (ESI) C_22_H_17_N_3_O_2_Cl_2_ [M+H]^+^: calcd. 426.0771, found 426.0767.

**3-methyl-1-(3-methylbenzyl)-1H-pyrazol-5-yl 3,7-dichloroquinoline-8-carboxylate (9c)**

White solid; yield, 76%, mp, 176.7-177.2 ℃; ^1^H NMR (400 MHz, DMSO, δ ppm): δ 9.04 (d, *J* = 2.4 Hz, 1H, Quinoline-H), 8.78 (d, *J* = 2.4 Hz, 1H, Quinoline-H), 8.21 (d, *J* = 8.8 Hz, 1H, Quinoline-H), 7.91 (d, *J* = 8.8 Hz, 1H, Quinoline-H), 7.23 (t, *J* = 7.6 Hz, 1H, Ar-H), 7.10 (d, *J* = 7.6 Hz, 1H, Ar-H), 6.99 (s, 1H, Ar-H), 6.91 (d, *J* = 7.6 Hz, 1H, Ar-H), 6.18 (s, 1H, CH), 5.21 (s, 2H, Ar-CH_2_), 2.28 (s, 6H, Ar-CH_3_,N-C-CH_3_). ^13^C NMR (100 MHz, DMSO, δ ppm): δ 163.05, 153.46, 151.88, 143.22, 140.88, 138.23, 137.49, 135.31, 131.55, 131.49, 131.39, 129.39, 129.13, 128.99, 128.60, 128.02, 127.16, 124.52, 96.29, 52.33, 21.47, 11.54. HRMS (ESI) C_22_H_17_N_3_O_2_Cl_2_ [M+H]^+^: calcd. 426.0771, found 426.0769.

**3-methyl-1-(4-methylbenzyl)-1H-pyrazol-5-yl 3,7-dichloroquinoline-8-carboxylate (9d)**

White solid; yield, 71%, mp, 162.2-163.5 ℃; ^1^H NMR (400 MHz, CDCl_3_, δ ppm): δ 8.87 (d, *J* = 2.4 Hz,1H, Quinoline-H), 8.14 (d, *J* = 2.4 Hz, 1H, Quinoline-H), 7.78 (d, *J* = 8.8 Hz, 1H, Quinoline-H), 7.58 (d, *J* = 8.8 Hz, 1H, Quinoline-H), 7.12 (d, *J* = 8.0 Hz, 2H, Ar-H), 7.05 (d, *J* = 8.0 Hz, 2H, Ar-H), 6.31 (s, 1H, CH), 5.19 (s, 2H, Ar-CH_2_), 2.31(s, 3H), 2.24 (s, 3H, N-C-CH_3_). ^13^C NMR (100 MHz, CDCl_3_, δ ppm): δ 163.07, 153.88, 151.27, 143.67, 140.00, 137.39, 133.78, 133.56, 132.27, 132.04, 129.52, 129.44, 129.40, 128.73, 126.90, 126.50, 96.54, 52.86, 21.11, 11.68. HRMS (ESI) C_22_H_17_N_3_O_2_Cl_2_ [M+H]^+^: calcd. 426.0771, found 446.0769.

**1-(2-chlorobenzyl)-3-methyl-1H-pyrazol-5-yl 3,7-dichloroquinoline-8-carboxylate (9e)**

White solid; yield, 63%, mp, 246.5-247.2 ℃; ^1^H NMR (400 MHz, DMSO, δ ppm): δ 8.95 (d, *J* = 2.8 Hz, 1H, Quinoline-H), 8.80 (d, *J* = 2.4 Hz, 1H, Quinoline-H), 8.25 (d, *J* = 9.2 Hz, 1H, Quinoline-H), 7.93 (d, *J* = 8.8 Hz, 1H, Quinoline-H), 7.32-7.34 (m, 2H, Ar-H), 7.20 (s, 1H, Ar-H), 7.11 (d, *J* = 6.4 Hz, 1H, Ar-H), 6.23 (s, 1H, CH), 5.27 (s, 2H, Ar-CH_2_), 2.21 (s, 3H, N-C-CH_3_). ^13^C NMR (100 MHz, DMSO, δppm): δ 161.78, 151.94, 147.63, 144.11, 143.27, 139.78, 135.49, 133.47, 131.98, 131.88, 130.88, 130.20, 129.54, 129.17, 127.91, 127.62, 127.20, 126.44, 94.79, 50.22, 14.63. HRMS (ESI) C_21_H_14_N_3_O_2_Cl_3_ [M+H]^+^: calcd. 446.0224, found 446.0227.

**1-(3-chlorobenzyl)-3-methyl-1H-pyrazol-5-yl 3,7-dichloroquinoline-8-carboxylate (9f)**

White solid; yield, 63%, mp, 139.9-141.2 ℃, ^1^H NMR (400 MHz, CDCl_3_, δ ppm): δ 8.74 (d, *J* = 2.4 Hz,1H, Quinoline-H), 8.16 (d, *J* = 2.4 Hz, 1H, Quinoline-H), 7.82 (d, *J* = 8.8 Hz, 1H, Quinoline-H), 7.61 (d, *J* = 8.8 Hz, 1H, Quinoline-H), 7.18-7.19 (m, 3H, Ar-H), 7.08-7.10 (m, 1H, Ar-H), 6.27 (s, 1H, CH), 5.24 (s, 2H, Ar-CH_2_), 2.31 (s, 3H, N-C-CH_3_). ^13^C NMR (100 MHz, CDCl_3_, δ ppm): δ 14.60, 50.59, 94.60, 125.63, 126.61, 127.60, 127.81, 128.87, 129.87, 129.89, 129.94, 131.02, 132.52, 133.91, 134.37, 138.69, 143.63, 144.47, 148.33, 151.40, 161.21. HRMS (ESI) C_21_H_14_N_3_O_2_Cl_3_ [M+H]^+^: calcd. 446.0224, found 446.0225.

**1-(4-chlorobenzyl)-3-methyl-1H-pyrazol-5-yl 3,7-dichloroquinoline-8-carboxylate (9g)**

Light yellow solid; yield, 72%, mp, 139.4-139.6 ℃; ^1^H NMR (400 MHz, DMSO, δ ppm): δ 8.94 (d, *J* = 2.8 Hz, 1H, Quinoline-H), 8.78 (d, *J* = 2.4 Hz, 1H, Quinoline-H), 8.24 (d, *J* = 8.8 Hz, 1H, Quinoline-H), 7.92 (d, *J* = 8.8 Hz, 1H, Quinoline-H), 7.31-7.34 (m, 2H, Ar-H), 7.19 (d, *J* = 3.2 Hz, 1H, Ar-H), 7.11 (d, *J* = 6.0 Hz, 1H, Ar-H), 6.22 (s, 1H), 5.27 (s, 2H, Ar-CH_2_), 2.21 (s, 3H, N-C-CH_3_). ^13^C NMR (100 MHz, DMSO, δ ppm): δ 161.77, 151.90, 147.63, 144.13, 143.27, 139.77, 135.44, 133.48, 131.94, 131.88, 130.84, 130.20, 129.53, 129.14, 127.90, 127.62, 127.17, 126.43, 94.78, 50.23, 14.63. HRMS (ESI) C_21_H_14_N_3_O_2_Cl_3_ [M+H]^+^: calcd. 446.0224, found 446.0227.

**3-methyl-1-(2-nitrobenzyl)-1H-pyrazol-5-yl 3,7-dichloroquinoline-8-carboxylate (9h)**

Gray solid; yield, 51%, mp, 189.4-190.5 ℃; ^1^H NMR (400 MHz, DMSO, δ ppm): δ 8.01 (d, *J* = 8.0 Hz, 1H, Quinoline-H), 7.92 (d, *J* = 8.0 Hz, 1H, Quinoline-H), 7.64 (t, *J* = 7.2 Hz, 2H), 7.56 (t, *J* = 8.4 Hz, 2H), 7.49 (t, *J* = 8.0 Hz, 1H), 7.42 (t, *J* = 7.6 Hz, 1H), 7.35 (d, *J* = 7.2 Hz, 2H), 6.02 (d, *J* = 8.6 Hz, 1H), 4.67 (s, 2H, Ar-CH_2_), 1.99 (s, 3H, N-C-CH_3_). ^13^C NMR (100 MHz, DMSO, δ ppm): δ 174.90, 161.67, 150.15, 148.01, 134.24, 133.46, 133.08, 131.42, 129.37, 129.28, 129.01, 128.59, 125.37, 125.33, 58.60, 44.06, 34.63,14.22. HRMS (ESI) C_21_H_14_N_4_O_4_Cl_2_ [M+H]^+^: calcd. 457.0464, found 457.0452.

**3-methyl-1-(3-nitrobenzyl)-1H-pyrazol-5-yl 3,7-dichloroquinoline-8-carboxylate (9i)**

Light yellow solid; yield, 55%, mp, 169.7-170.4℃, ^1^H NMR (400 MHz, DMSO, δ ppm): δ 8.01 (t, *J* = 8.0 Hz, 1H, Quinoline-H), 7.93 (s, 2H), 7.68 (s, 1H), 7.56 (d, *J* = 7.6 Hz, 2H), 7.48 (t, *J* = 8.0 Hz, 1H), 7.32 (t, *J* = 8.0 Hz, 1H), 6.93 (d, *J* = 8.0 Hz, 1H), 4.47 (s, 2H, Ar-CH_2_), 2.25 (s, 3H, N-C-CH_3_). ^13^C NMR (100 MHz, DMSO, δ ppm): δ 14.91, 38.98, 45.98, 60.98, 122.54, 122.66, 124.21, 129.99, 130.17, 134.21, 136.52, 137.58, 138.75, 147.79, 147.91, 161.00, 174.80. HRMS (ESI) C_21_H_14_N_4_O_4_Cl_2_ [M+H]^+^: calcd. 457.0464, found 457.0456.

**1,3-dimethyl-1H-pyrazol-5-yl 3,7-dichloroquinoline-8-carboxylate (10a)**

White solid; yield, 78%, mp, 146.2-146.6 ℃, ^1^H NMR (400 MHz, CDCl_3_, δ ppm): δ 8.87 (d, *J* = 1.6 Hz, 1H, Quinoline-H ), 8.20 (d, *J* = 1.6 Hz, 1H, Quinoline-H), 7.86 (d, *J* = 8.8 Hz, 1H, Quinoline-H), 7.65 (d, *J* = 7.6 Hz, 1H, Quinoline-H), 6.17 (s, 1H, CH), 3.79 (s, 3H N-CH_3_), 2.30 (s, 3H, N-C-CH_3_). ^13^C NMR (100 MHz, CDCl_3_, δ ppm): δ 14.43, 34.65, 94.27, 126.58, 128.82, 129.90, 131.09, 132.43, 133.93, 143.63, 144.46, 147.32, 151.40, 161.51. HRMS (ESI) C_15_H_11_N_3_O_2_Cl_2_ [M+H]^+^: calcd. 336.0301, found 336.0302.

**1-ethyl-3-methyl-1H-pyrazol-5-yl 3,7-dichloroquinoline-8-carboxylate (10b)**

Light yellow solid, 69%; yield, mp, 103.1-104.8 ℃; ^1^H NMR (400 MHz, DMSO, δ ppm): δ 9.04 (d, *J* = 2.4 Hz, 1H, Quinoline-H), 8.76 (d, *J* = 2.4 Hz, 1H, Quinoline-H), 8.20 (d, *J* = 8.8 Hz, 1H, Quinoline-H), 7.89 (d, *J* = 8.8 Hz, 1H, Quinoline-H), 6.11 (s, 1H, CH), 3.98-4.03 (m, 2H, CH_3_-CH_2_), 2.31 (s, 3H, N-C-CH_3_), 1.29 (t, *J* = 7.6 Hz, 3H, N-CH_2_-CH_3_). ^13^C NMR (100 MHz, DMSO, δ ppm): δ 163.02. 153.16, 151.85, 143.24, 139.88, 135.28, 131.61, 131.54, 131.32, 129.39, 129.12, 127.16, 95.64, 43.66, 15.46, 11.22. HRMS (ESI) C_16_H_13_N_3_O_2_Cl_2_ [M+H]^+^: calcd. 350.0458, found 350.0458.


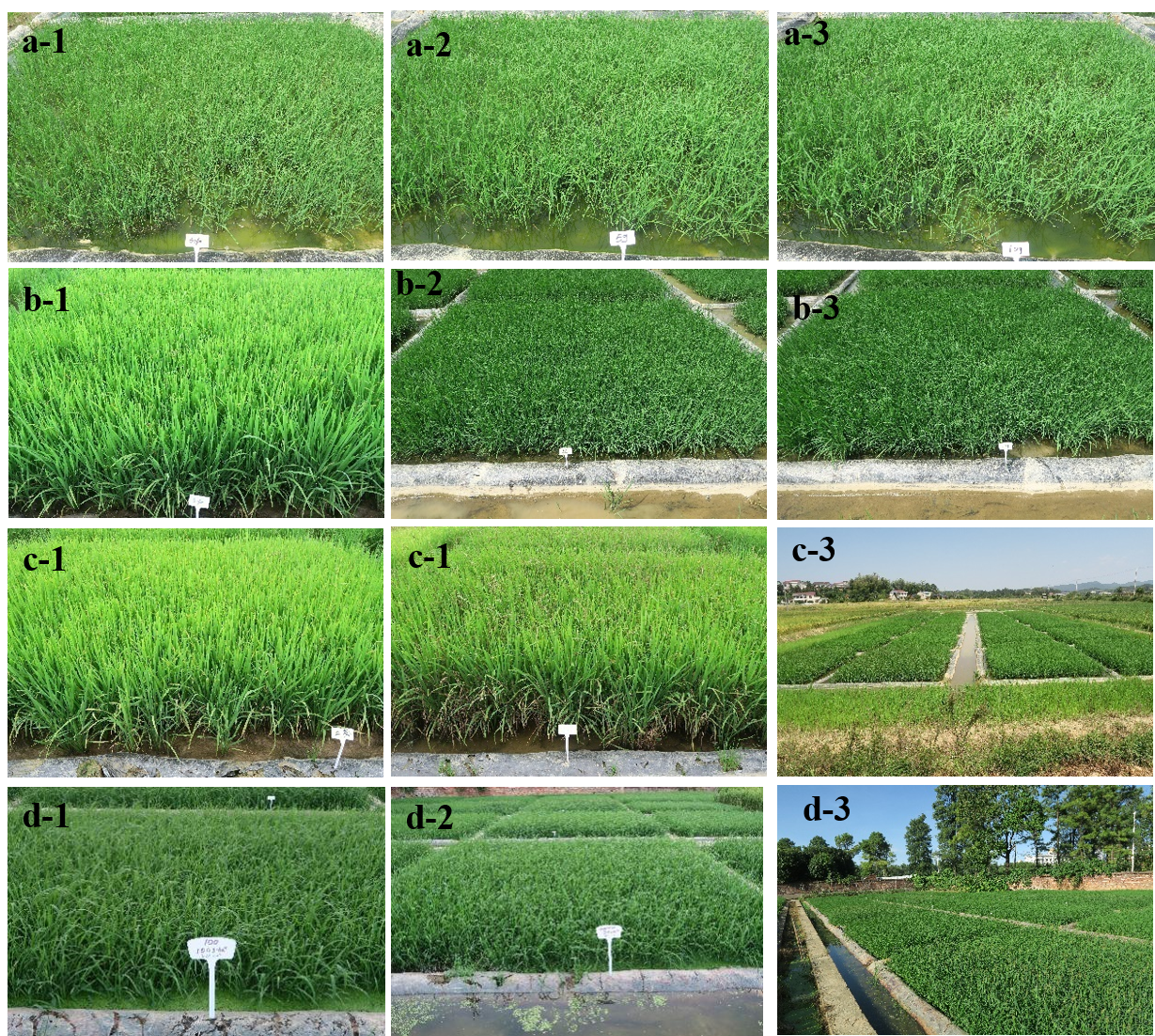


**Supplementary Figure S1.** Herbicidal activity of compound **10a** in the rice paddy in 2019 and 2020. a-1, a-2 and a-3 were treated **10a** dosage of 45,75,150 g a.i. / ha after 14 days. **b-1**, **b-2** and **b-3** were treated **10a** dosage of 45, 75, 150 g a.i. / ha after 21 days. **c-1**, **c-2** and c**-3** were sprayed 300 g a.i. / ha quinclorac and control blank after 52 days. **c-3** was the full views of the **10a** field weeding efficacy trial in 2019. **d-1** and were treated **10a** dosage of 150 g a.i. / ha and **d-2** sprayed 300 g a.i. / ha quinclorac after 14 days in 2019. **d-3** was the full views of the **10a** field weeding efficacy trial in 2020.

**Table 3.** Weed control effects after 14, 21days treated different dosage of **10a** and quinclorac.

| Treatment  g / ha | Barnyard grass (control effect %) | | | | Total weeds (control effect %) | | | |
| --- | --- | --- | --- | --- | --- | --- | --- | --- |
|  | 2019 | | 2020 | | 2019 | | 2020 | |
|  | 14 d | 21 d | 14 d | 21 d | 14 d | 21 d | 14 d | 21 d |
| 45 (**10a**) | 39.7  38.5  43.9 | 49.5  48.6  59.1 | 41.1  47.8  42.2 | 48.1  52.8  50.9 | 44.4  41.1  34.8 | 46.3  45.9  44.6 | 38.1  38.1  40.2 | 40.7  46.8  47.2 |
| 75 (**10a**) | 63.8  66.1  65.4 | 74.2  77.2  76.9 | 66.4  71.7  68.6 | 71.8  76.2  74.0 | 65.3  65.8  64.5 | 71.6  72.5  67.1 | 60.7  60.1  66.1 | 67.7  67.5  69.1 |
| 120 (**10a**) | 74.9  76.5  84.4 | 81.8  85.1  86.3 | 78.3  79.9  81.2 | 81.6  83.2  85.4 | 77.5  77.0  78.9 | 81.8  81.2  82.7 | 78.8  78.6  79.3 | 83.6  82.3  83.7 |
| 150 (**10a**) | 89.6  89.9  88.1 | 94.1  95.5  93.9 | 87.5  91.1  90.2 | 93.2  93.9  93.4 | 88.4  90.4  90.3 | 90.1  93.5  91.2 | 92.2  93.1  92.8 | 94.5  95.9  95.2 |
| 300 (**Q**) | 93.3  89.2  92.9 | 95.1  93.4  93.8 | 88.5  90.4  91.7 | 90.4  95.2  95.8 | 87.5  91.4  91.1 | 91.5  93.2  92.2 | 90.7  92.8  91.9 | 93.1  94.8  94.1 |


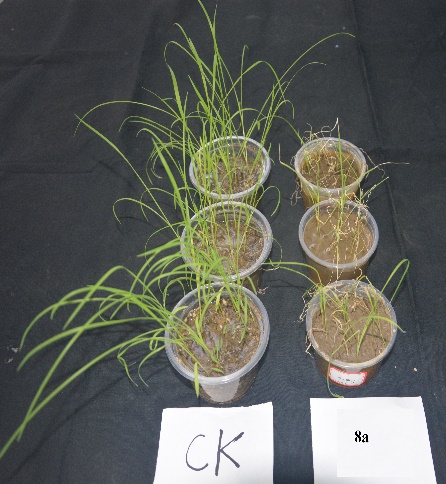

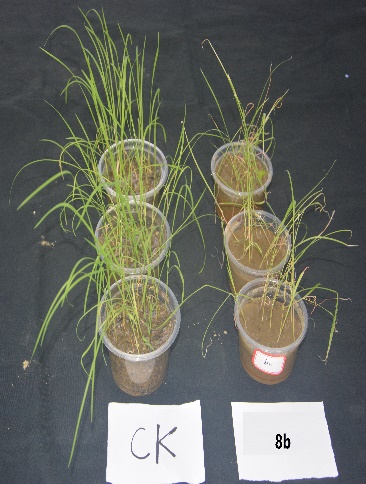

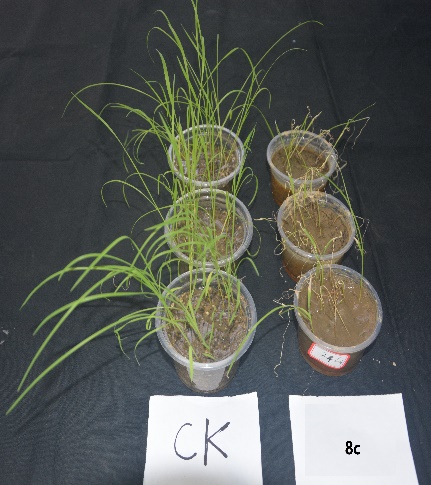


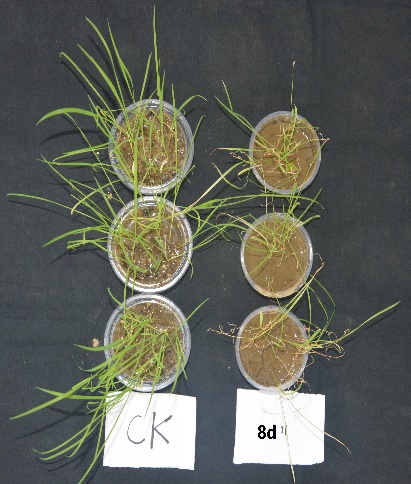

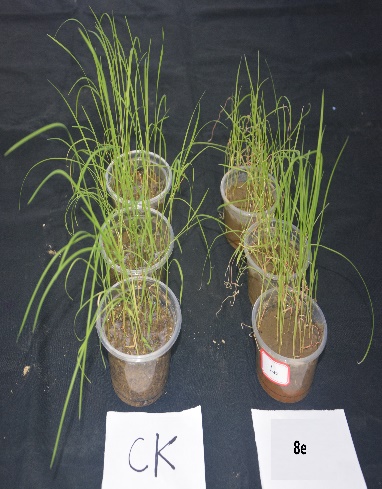

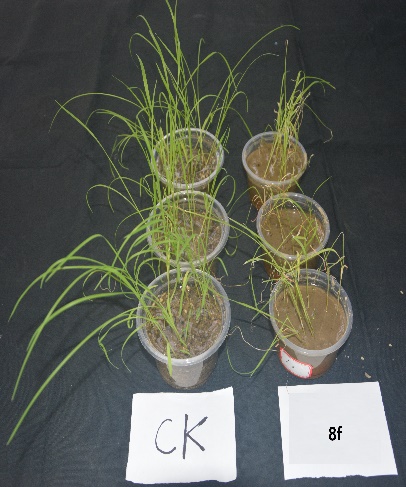


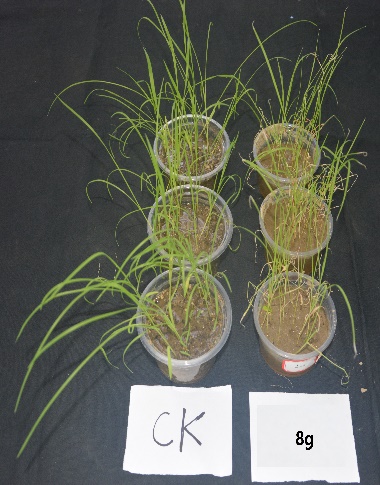

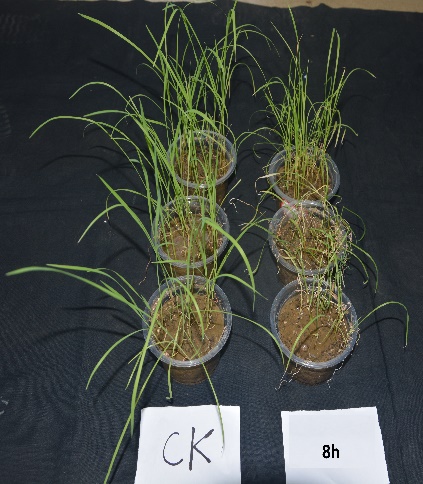

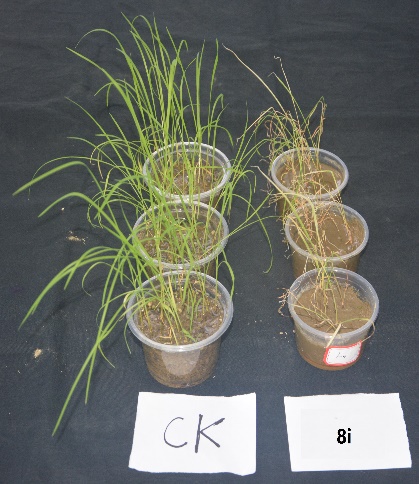


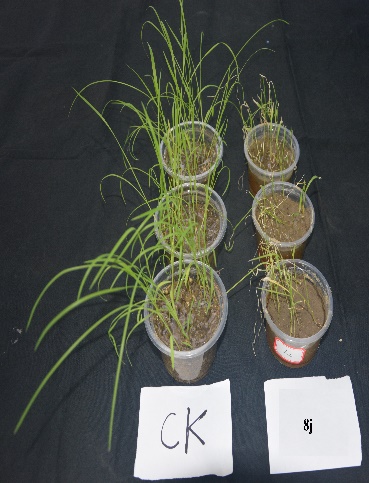

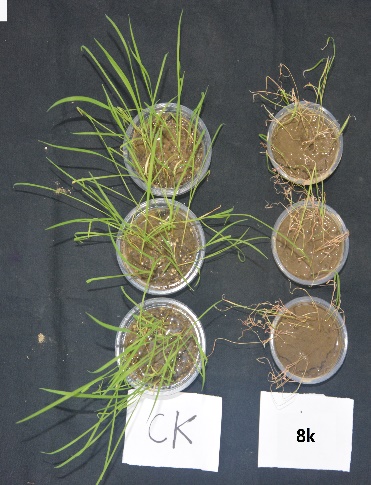

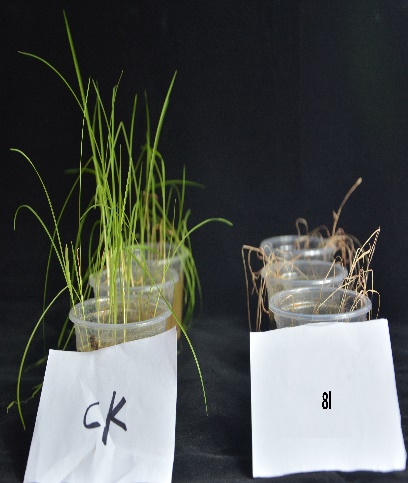


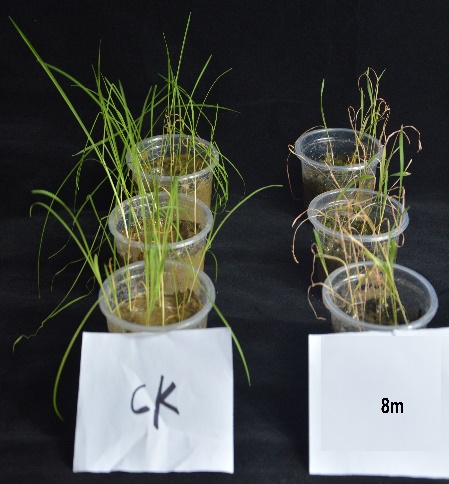

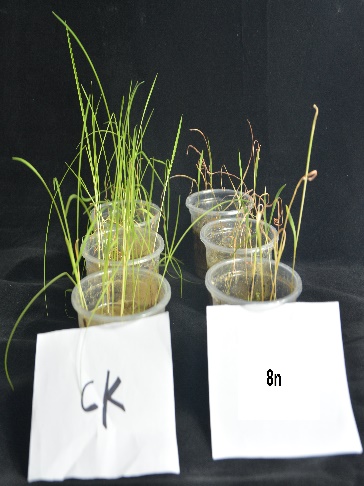

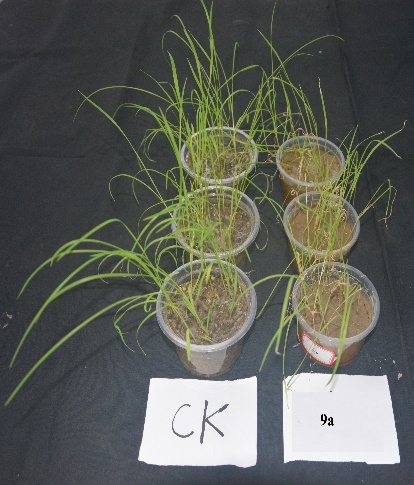


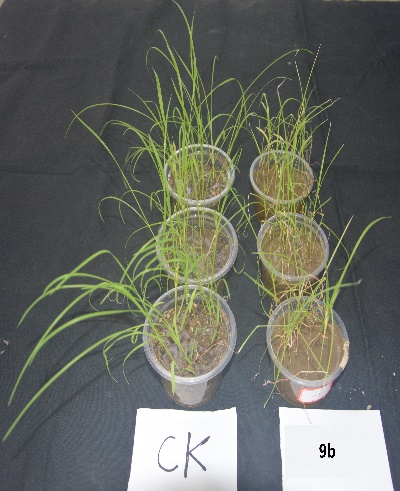

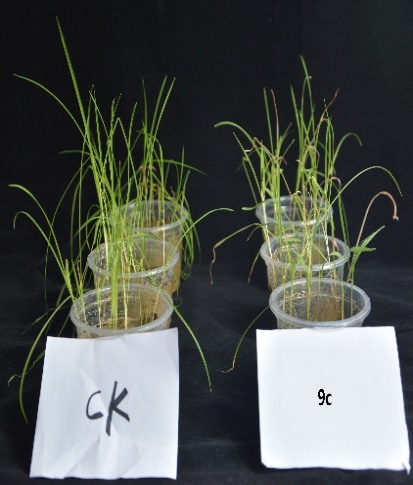

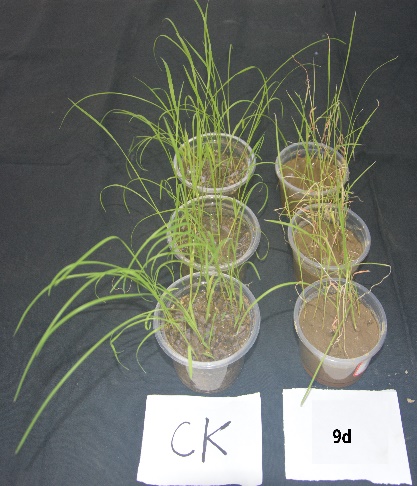


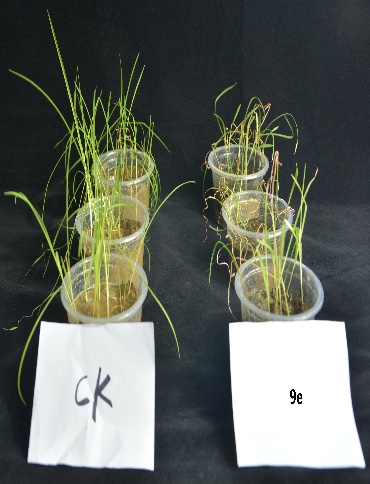

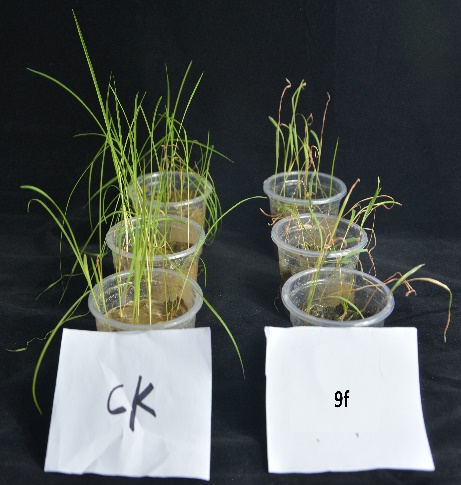

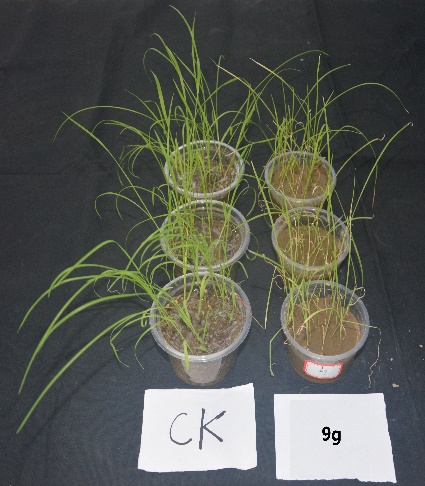


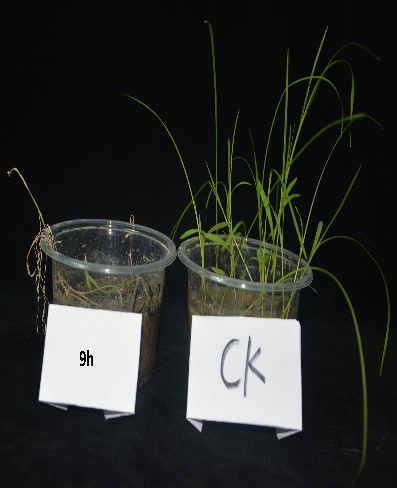

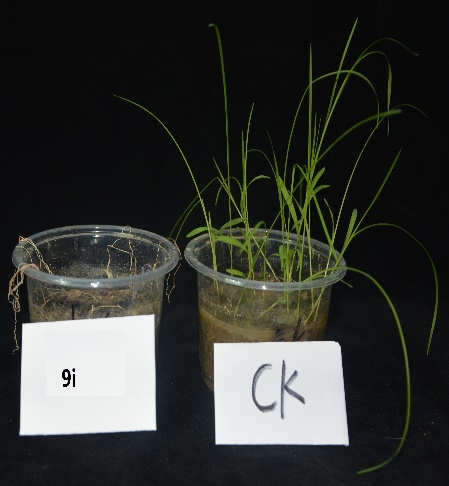

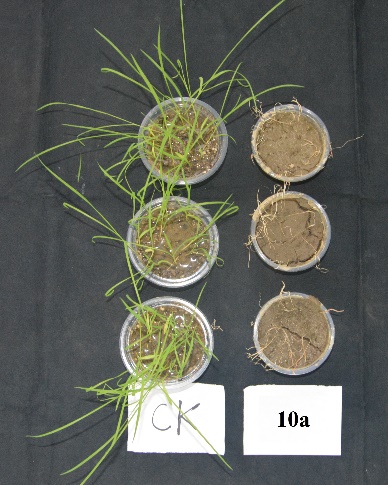


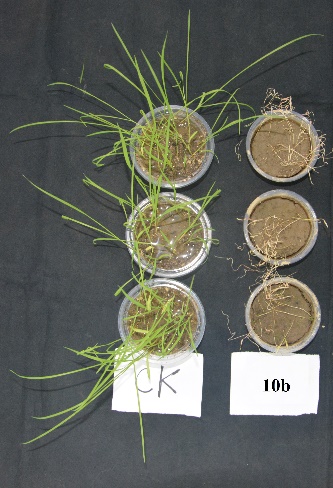


**Supplementary Figure S2.** Herbicidal activity of barnyard grass treated 375 g a.i. / ha compounds **8a-n**, **9a-i** and **10a-b** after 14 days in greenhouse
